# Negative feedback regulation by HuR controls TRIM21 expression and function in response to UV radiation

**DOI:** 10.1038/s41598-020-68646-3

**Published:** 2020-07-16

**Authors:** Abhishek Guha, Sharanya Nag, Partho Sarothi Ray

**Affiliations:** 1 Department of Biological Sciences, Indian Institute of Science Education and Research Kolkata, Mohanpur, West Bengal 741246 India; 2 Present Address: Department of Neurology, University of Alabama, Birmingham, AL 35294 USA

**Keywords:** Cancer, Cell biology, Molecular biology

## Abstract

The E3 ubiquitin ligase TRIM21 plays a crucial role as a negative regulator of innate immune responses. Recent evidence has also indicated the involvement of TRIM21 in the genotoxic stress response and suppressing tumorigenesis. Our previous work has demonstrated a new function of TRIM21 in inhibiting p53 protein synthesis by degrading the RNA-binding protein HuR in response to UV radiation. This suggested a pro-oncogenic role of TRIM21. In this study, we have shown that TRIM21 enhances the proliferation of MCF7 breast carcinoma cells and counteracts the decrease in cell proliferation and colony formation caused by UV-induced DNA damage. Further, this pro-oncogenic role of TRIM21 in response to DNA damage is mediated by its degradation of HuR. Conversely, we found that HuR binds to a U-rich element in the 3′UTR of *TRIM21* mRNA and activates its translation, thereby constituting a negative feedback loop. We found that dihydrotanshinone-I (DHTS-I), a plant-derived product which prevents HuR binding to specific RNAs, prevented HuR-mediated upregulation of TRIM21, while increasing the HuR-mediated upregulation of p53. Together, these findings demonstrate a negative feedback regulation between TRIM21 and HuR, which may play an important role in regulating the level of p53 in the genotoxic stress response.

## Introduction

TRIM (Tripartite motif) family proteins are ubiquitously found in all metazoans and are recognized for conserved biological functions, such as control of cell proliferation and differentiation. However, with the appearance of complex immune systems in jawed vertebrates, some of these have evolved to directly regulate antiviral action and cytokine production. TRIM family proteins are primarily recognized as post-translational modifiers. Nevertheless, some of the members of this family are implicated in RNA metabolism. These TRIM proteins are specifically localized in the cytoplasmic processing bodies. Furthermore, about half of all the TRIM family proteins are involved in autophagy. Most of these are also involved in the formation of TRIMosomes which are considered to facilitate autophagic engulfment and subsequent lysosomal degradation^1^.

E3 ubiquitin ligase TRIM21 is a 52 kDa protein belonging to the TRIM family. Similar to many other TRIM family proteins, TRIM21 has a characteristic N-terminal RING domain, B-box domain, coiled-coil domain (collectively called RBCC). The RING domain is implicated in E3 ubiquitin ligase activity, whereas the B-box domain has been shown to be a negative regulator of RING domain. Recently, the phosphorylation of serine-80 (S80) or phosphomimetic serine-80 to glutamate (S80E) mutation in TRIM21 has been found to release the inhibition of B-box domain^2,3^. The coiled-coil domain is utilized in homo-dimerization of TRIM21 which ensures binding with the substrate in a stoichiometry of 2:1^2^. TRIM21 has also C-terminal PRYSPRY domain which is required for target protein recognition. Structural analysis of PRYSPRY domain of TRIM21 has demonstrated its diverse functions. Isothermal titration calorimetric (ITC) study has revealed that two molecules of TRIM21 bind to each IgG Fc with a *K*_*d*_ of 37nM^4^. Evidences have also demonstrated that this interaction is conserved among different mammalian species^4^.

TRIM21 is an autoantigen which is found in the sera of patients with systemic lupus erythematosus (SLE), rheumatoid arthritis, and Sjögren’s syndrome. It is therefore also referred to as Ro52/SSA and is shown to be conserved among mammalian species^5,6^. In presence of E2 enzyme UbcH5, TRIM21 is auto-ubiquitinated at lysine-48 (K48), whereas by utilizing E2 pair Ubc13/UEV1A, TRIM21 undergoes auto-ubiquitination at lysine-63 (K63). These are reported to be the important modifications for TRIM21 action^7^. TRIM21 has been recognized as a cytoplasmic Fc receptor which mediates antibody-dependent intracellular neutralization of viral particles by triggering proteasomal degradation^8^. In addition, during viral or bacterial infection, TRIM21 stimulates a number of transcription factors, such as NF-κB, AP-1 and IRF, thereby controlling the production of proinflammatory cytokines^9^. TRIM21 is also found to be phosphorylated at tyrosine-393 (Y393) in PRYSPRY domain in response to TLR stimulation which is shown to be critical for interaction with IRF3 and IFN-β production^10^. Additionally, it is reported to negatively regulate innate immune responses by controlling IRF3-mediated IFN-β production in response to Japanese Encephalitis virus (JEV) infection^11^. TRIM21 has also been shown to degrade IRF3 by autophagy and suppresses IFN response^12^. Moreover, it is involved in differential stability of IRF5 isoforms following TLR7 stimulation^13^. TRIM21 enhances IL-2 production in human immortalized T cells in response to CD28 stimulation^14^. It is also involved in Th1/Th17 differentiation through secretion of proinflammatory cytokines^15^. Furthermore, T-cell proliferation and IL6 production have been found to be regulated by TRIM21^16^. TRIM21 is also shown to inhibit innate immune response by controlling DDX41 degradation in response to intracellular dsDNA virus infection^17^. Knockout studies have revealed that TRIM21 is a negative regulator of proinflammatory cytokine production and IFN signalling^18,19^. TRIM21 deficient mice were also reported to be highly susceptible to *Toxoplasma gondii* infection^20^. Therefore, the role of TRIM21 has been well expounded in the area of innate immune response. However, TRIM21 has further been implicated in neurodegenerative disorders, such as Alzheimer’s disease. TRIM21 is reported to prevent tau (τ) aggregation, thereby reducing the propensity for Alzheimer’s disease^21^. It also interacts with human decapping enzyme 2 (hDCP2) and enhances decapping activity in P-bodies, thereby regulating mRNA metabolism^22^. Moreover, TRIM21 is also thought to play a role in different human cancers. It has been found to be downregulated in many liver cancer patients^23^. Moreover, TRIM21 is involved in the epithelial to mesenchymal transition in breast cancer cells^24^. TRIM21 also promotes the proliferation of osteosarcoma cells and its expression enhances the tolerance of osteosarcoma cells to various stresses^25^. It also affects the expression and stability of p53 in different cancers^26–28^. Recently, TRIM21 has been shown to cause the temporally-regulated degradation of the RNA-binding protein HuR in response to UVC irradiation. The negative regulation of HuR by TRIM21 contributes to the pulsatile change in p53 protein level in response to UV irradiation, thereby limiting the expression of p53 under DNA damage^26^. However, the physiological role of TRIM21 in response to DNA damage has not been elucidated.

TRIM21 is found to be upregulated in many autoimmune disorders including SLE and Sjögren’s syndrome^5^. JEV infection in human microglial cells has also been reported to induce TRIM21 expression in a time dependent manner^11^. TRIM21 has been shown to be induced by IFN-α and IFN-β. TRIM21 expression is found to be positively induced by both IRF-1 and IRF2, whereas IRF-4 and IRF-8 are shown to act as repressors for TRIM21 transcription^29^. TRIM21 has also been found to be downregulated at posttranscriptional levels by microRNA miR-494-3p in breast cancer cells^30^. However, the regulatory mechanisms that control TRIM21 expression are still unresolved.

Here, we report an increase in the proliferation and colony formation of breast cancer cells as a result of TRIM21 expression. TRIM21 overexpression was also found to reverse the effect of UV irradiation on cell proliferation and colony formation, thereby demonstrating a pro-oncogenic function of TRIM21 that was mediated by its regulation of HuR. Furthermore, HuR was also shown to interact with the 3′UTR of *TRIM21* mRNA both in vitro and in cells. In addition, HuR overexpression was found to activate the translation of *TRIM21* mRNA in a *TRIM21* mRNA 3′UTR-dependent manner. This constituted a negative feedback regulation between TRIM21 and HuR in response to genotoxic stress.

## Results

### TRIM21 reverses the decrease in cell proliferation and colony formation caused by UV irradiation

**Figure 1:**
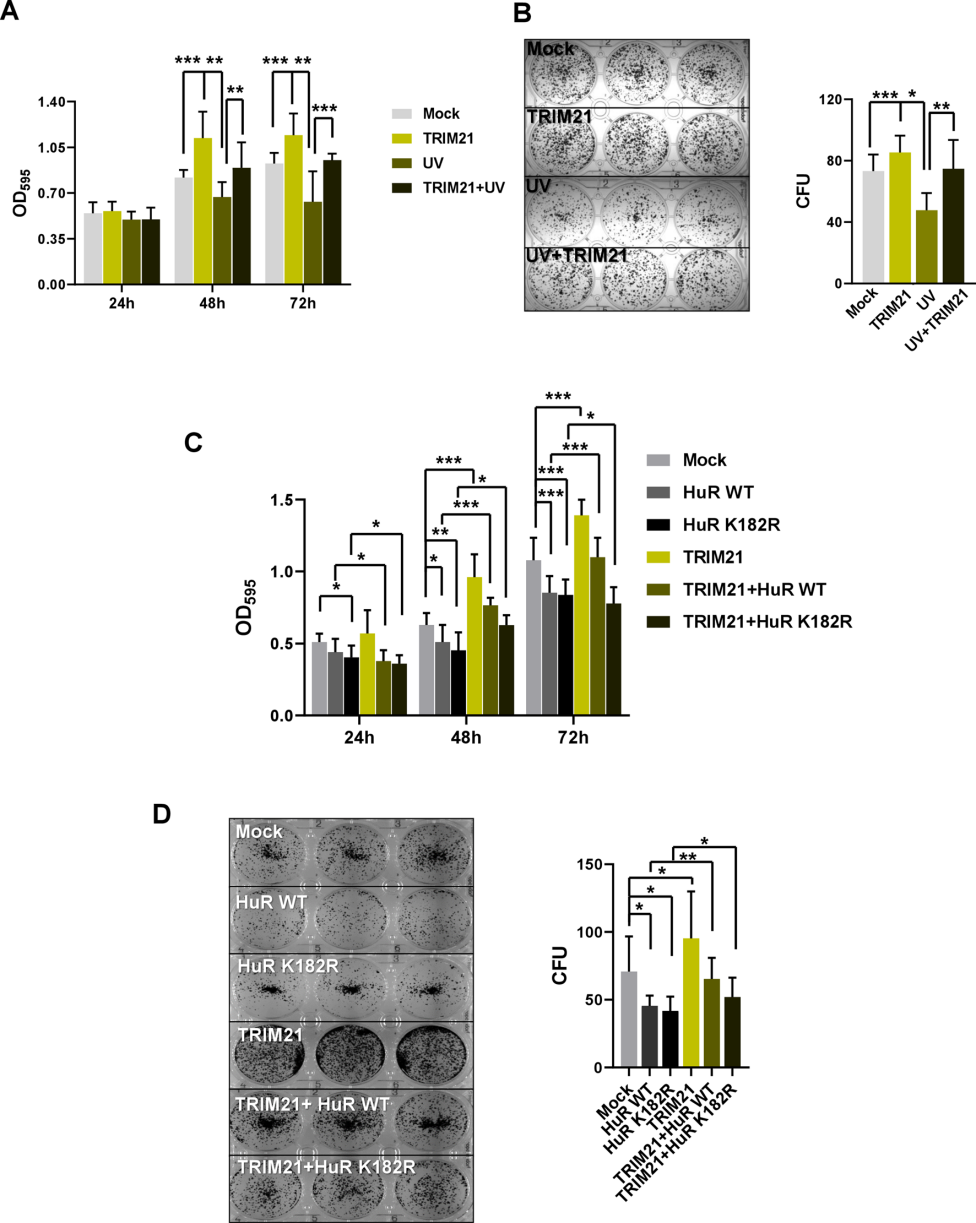
TRIM21 reverses the decrease in cell proliferation and colony formation caused by UV irradiation. (**A**) MCF7 cells transfected with TRIM21-expressing plasmid construct were exposed to 10 J/m^2^ of UVC radiation or not exposed, and allowed to grow for 72 h post treatment. Cells were harvested and MTT assay was performed at indicated time points. (**B**) MCF7 cells exposed to UV were seeded and colonies were counted after 12–14 days by crystal violet staining (Left panel). Normalized CFUs from three independent experiments were plotted (Right panel). (**C**) MCF7 cells transfected with different plasmid constructs expressing TRIM21 and/or HuR were allowed to grow post transfection. MTT assay was performed at indicated time points. (**D**) Transfected MCF7 cells as indicated in C were seeded and colonies were counted after 12–14 days by crystal violet staining (Left panel). Normalized CFUs from three independent experiments were plotted (Right panel). *Signifies a *p* value ≤ 0.05, **signifies a *p* value ≤ 0.01, ***signifies a *p* value ≤ 0.005 (paired two-tailed or one-tailed t-test).

As TRIM21 has been shown to interact with HuR and mediates ubiquitination-dependent proteasomal degradation of HuR under genotoxic stress^26^, we sought to understand in more detail the cellular function of TRIM21 in the context of DNA damage. We found that TRIM21 knockdown abrogates the decrease of HuR post 4 h of UV exposure (Fig. S1). Furthermore, the increase in HuR level observed till 4 h post UV irradiation is also reduced upon TRIM21 overexpression (Fig. S2), thereby reinforcing the role of TRIM21 as a negative regulator of HuR in response to UV irradiation. As HuR is a positive regulator of the tumor suppressor p53, we therefore investigated the effect of TRIM21 on cancer cell proliferation and colony formation. Overexpression of TRIM21 in MCF7 breast carcinoma cells increased cell proliferation and colony formation (Fig. 1A,B). However, the proliferation and colony forming potential of MCF7 cells were significantly reduced due to UV treatment. When TRIM21 expressing MCF7 cells were UV irradiated, TRIM21 was found to significantly reverse the decreased cell proliferation and colony formation caused by UV induced DNA damage (Fig. 1A,B). We then checked whether the pro-proliferative function of TRIM21 was mediated via its degradation of HuR. HuR overexpression has been shown to reduce cell proliferation and colony formation, both in absence and presence of UV irradiation^31^. Therefore, HuR WT and HuR K182R (lysine 182 to arginine mutation), which is refractory to TRIM21-mediated degradation, were overexpressed in MCF7 cells in absence and presence of TRIM21 overexpression. HuR overexpression reduced cell proliferation and colony formation as observed before, and the K182R mutant HuR caused an even greater reduction. TRIM21 overexpression significantly enhanced the cell proliferation and colony formation, but this was partly reduced on expression of WT HuR and more significantly reduced on expression of K182R mutant HuR which is not degraded by TRIM21 (Fig. 1C,D). Together, these findings demonstrated a pro-oncogenic role of TRIM21 under DNA damage condition, which is, at least partly, mediated by its degradation of HuR.

### HuR increases TRIM21 protein level in breast cancer cells

**Figure 2:**
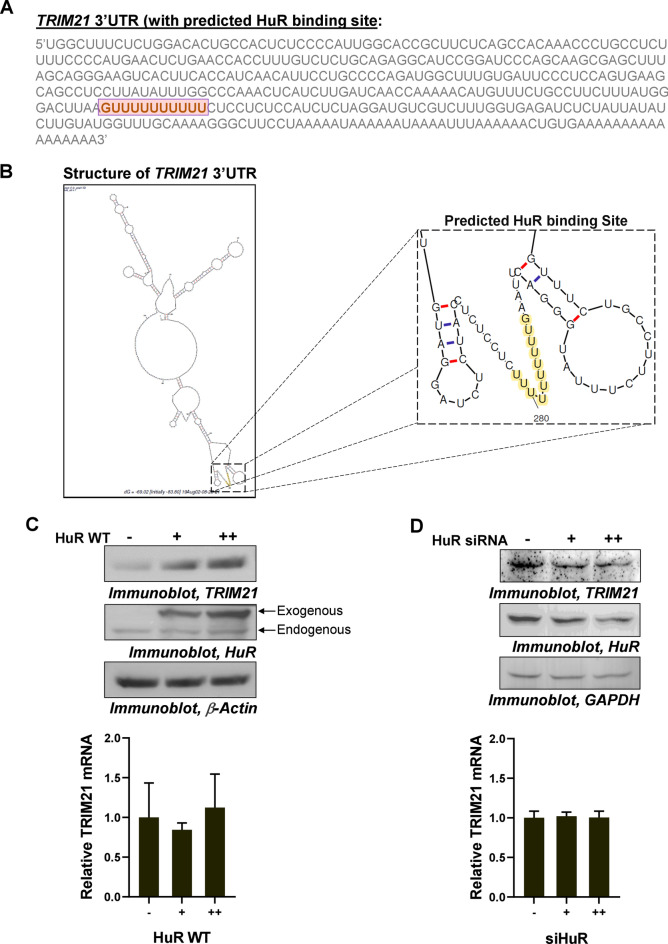
Posttranscriptional regulation of TRIM21 by HuR. (**A**) Sequence of 3′UTR of *TRIM21 mRNA* indicating a putative U-rich HuR binding site. (**B**) mFold structure of 3′UTR of *TRIM21* mRNA including HuR binding site (highlighted in yellow). (**C**) Immunoblots of lysates of MCF7 cells transfected with two increasing concentrations of Myc-tagged-HuR (WT) expressing constructs probed with TRIM21, HuR, and β-Actin antibodies (upper panel). qRT-PCR of total RNA from the same cell lysate with TRIM21- and GAPDH-specific primers (lower panel). (**D**) Immunoblots of lysates of MCF7 cells transfected with two increasing concentrations of siRNA against endogenous *HuR* probed with TRIM21, HuR, and GAPDH antibodies (upper panel). qRT-PCR of total RNA from the same cell lysate with TRIM21- and GAPDH specific primers (lower panel). Mean ± SD from three independent experiments, each with two technical replicates are represented in all graphs.

As we demonstrated that the physiological and molecular function of TRIM21 under DNA damage condition is mediated by its regulation of HuR, we sought to investigate whether HuR reciprocally plays any role in the regulation of TRIM21 expression. In order to investigate this, we utilized available PAR-CLIP data for HuR binding to mRNA and RBP binding site prediction algorithms to analyse *TRIM21* mRNA 3′UTR for presence of putative HuR binding site(s). PAR-CLIP data from multiple studies^32–35^ showed a region between 260 and 300 nt of the *TRIM21* mRNA 3′UTR as HuR-binding region (Fig. S3). The binding site prediction algorithms^36,37^ predicted a poly(U) stretch within this region as the putative HuR binding site in the 3′UTR of *TRIM21* mRNA (Fig. 2A and Fig. S4). Taking this as the minimal HuR-binding sequence, mFold algorithm was used to predict the structure of *TRIM21* mRNA 3′UTR which showed the presence of this HuR binding sequence in a single-stranded region (Fig. 2B). This also supported our hypothesis as HuR has been shown to be a single stranded RNA-binding protein. HuR was overexpressed in MCF7 cells which strongly enhanced TRIM21 protein levels in a dose-dependent manner, keeping the mRNA level unaltered (Fig. 2C and Fig. S10). The siRNA-mediated knockdown of cellular HuR in MCF7 cells also caused dose-dependent decrease of TRIM21 protein level, without affecting the mRNA levels (Fig. 2D and Fig. S11). Similar phenomenon was observed in case of the highly aggressive triple-negative breast cancer cell line MDA-MB-231 (Fig. S5). In summary, these results indicated a posttranscriptional regulation of TRIM21 mediated via HuR.

### Overexpression of HuR in breast cancer cells enhances the translation of *TRIM21* mRNA

**Figure 3:**
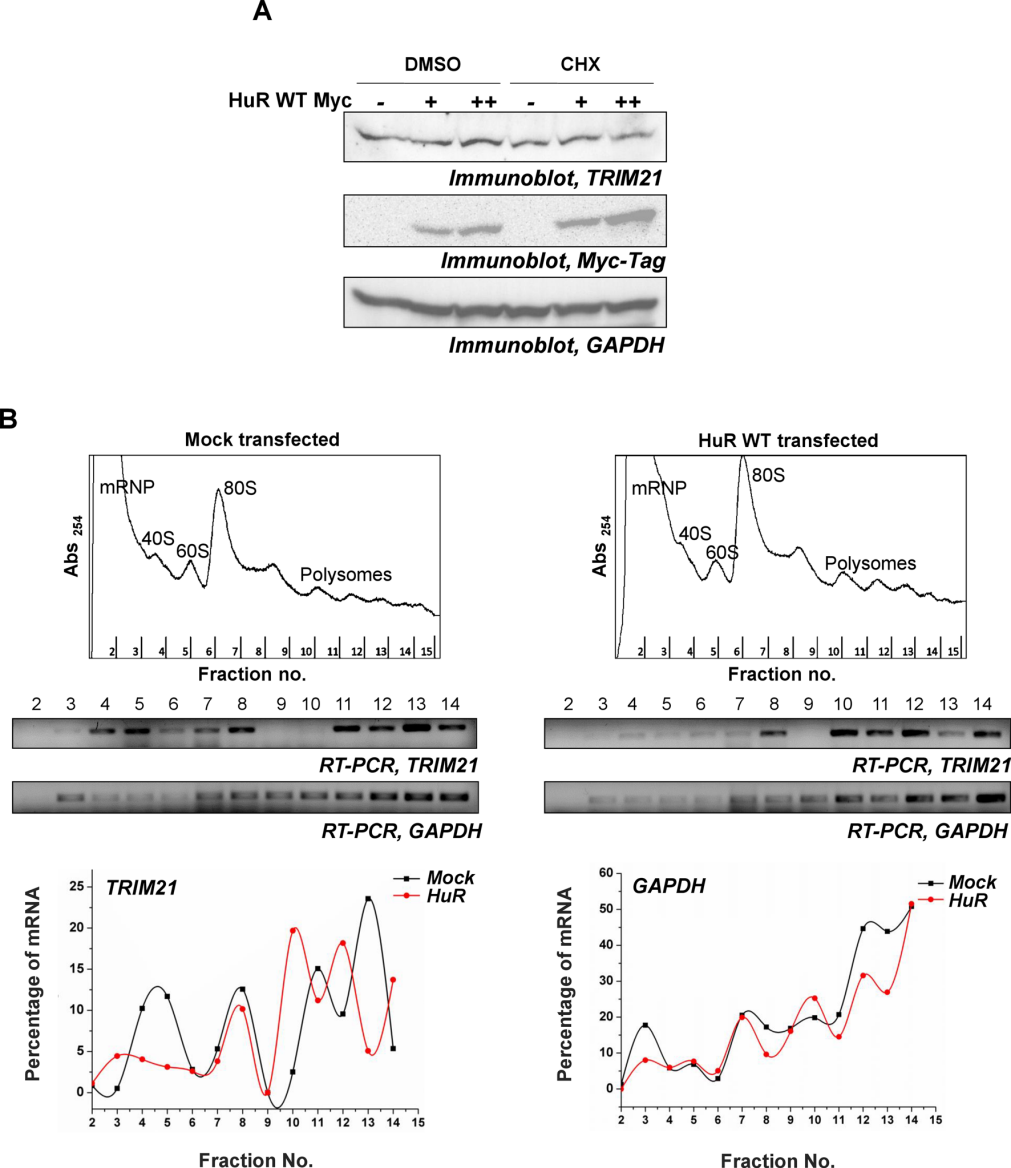
HuR enhances the translation of *TRIM21* mRNA. (**A**) MCF7 cells transfected with two increasing concentrations of Myc-tagged-HuR (WT) expressing constructs were treated with or without CHX. Immunoblotting was performed from the lysates using TRIM21, Myc-tag, and GAPDH specific antibodies. (**B**) Ribosomal fractions from MCF7 cells, either mock transfected or transfected with Myc-tagged HuR (WT) expressing constructs, were analysed by sucrose density gradient fractionation (upper panels). Total RNAs isolated from the indicated fractions were analysed by semi-quantitative RT-PCR using TRIM21 and GAPDH primers (middle panels). Percentage of band intensity was plotted against the fraction number (lower panels).

In order to determine whether overexpression of HuR enhances *TRIM21* mRNA translation, the “translational index” was calculated, which measures the ratio of protein levels relative to the corresponding mRNA levels (Fig. S6). The value of the index was significantly increased (from 1 to ~ 2.3) with the overexpression of HuR, supporting the notion that the translation of *TRIM21* was enhanced by HuR (Fig. S6). In agreement with this finding, CHX treatment of MCF7 cells was found to prevent the upregulation of TRIM21 protein in presence of HuR overexpression (Fig. 3A and Fig. S12). Further, to directly test the phenomenon of translation upregulation, polysome profiling was carried out with the control and HuR expressing MCF7 cells. Analysis of ribosomal fractions from control cells showed that *TRIM21* mRNA was present in both lighter non-translating and heavier translating ribosomal fractions. However, in HuR expressing cells the *TRIM21* mRNAs completely shifted to the heavier translating fractions. These changes were not observed when the distribution of *GAPDH* mRNA was tested under both the conditions (Fig. 3B and Fig. S13). Together, these data demonstrated that HuR enhances the translation of *TRIM21* mRNA.

### HuR binds to the 3′UTR of *TRIM21* mRNA in cells and in vitro

**Figure 4:**
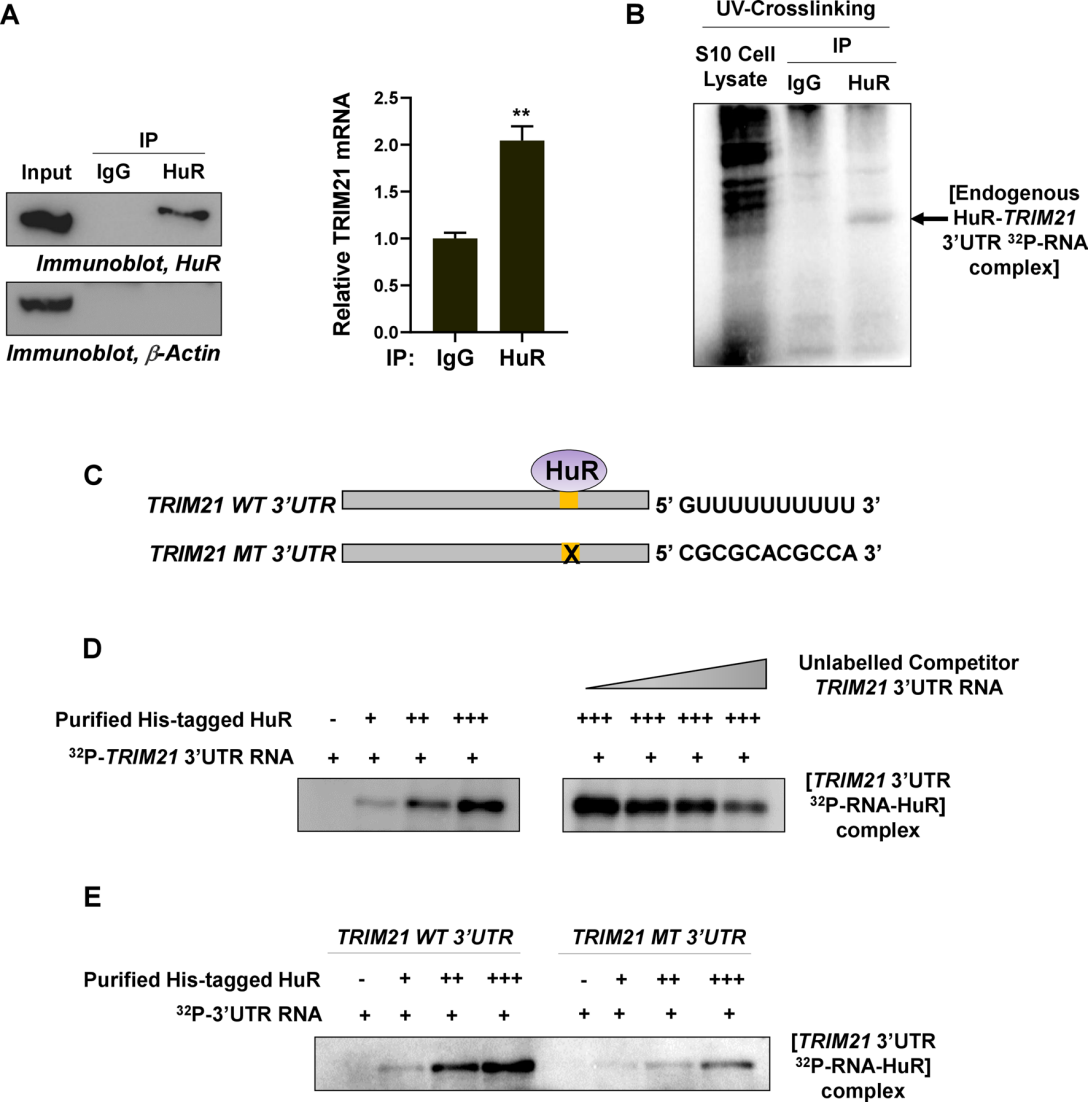
HuR interacts with 3′UTR of *TRIM21* mRNA in cells and in vitro. (**A**) MCF7 cell lysates were immunoprecipitated using nonspecific and HuR-specific antibodies and immunoblotted using HuR and β-Actin antibodies (left panel). qRT-PCR from the total RNA isolated from the immunoprecipitated samples was performed using TRIM21 and GAPDH specific primers (Right panel). Data represents mean ± SD values from 3 independent experiments. **Signifies a *p* value ≤ 0.01, (paired two-tailed t-test). (**B**) ^32^P-labelled 3′UTR of *TRIM21* mRNA was incubated with MCF7 S10 cytoplasmic lysate, UV-crosslinked and RNaseA digested. The RNA–protein complexes were immunoprecipitated using HuR-specific and non-specific antibodies and electrophoresed on 12% SDS-PAGE gel. (**C**) Schematic diagram of the *TRIM21* wild-type (*TRIM21* WT) and *TRIM21* mutant (*TRIM21* MT) 3′UTR RNAs. (**D**) ^32^P-labelled 3′UTR of *TRIM21* mRNA was incubated with three increasing concentrations of purified his-tagged HuR, UV-crosslinked and RNaseA digested. The RNA–protein complexes were resolved on 12% SDS-PAGE gel (left panel). Purified HuR was incubated with three increasing concentrations of unlabelled TRIM21 3′UTR prior to the incubation with ^32^P-labelled *TRIM21* 3′UTR. UV-crosslinking was done followed by RNaseA digestion and RNA–protein complexes were resolved using 12% SDS-PAGE gel (Right panel). (**E**) ^32^P-labelled *TRIM21* WT and MT 3′UTRs were incubated with three increasing concentrations of purified his-tagged HuR, UV-crosslinked and RNaseA digested. The RNA–protein complexes were resolved on 12% SDS-PAGE gel.

As we deduced a HuR binding site in the 3′UTR of *TRIM21* mRNA using PAR-CLIP data and binding site prediction algorithms, we sought to validate the interaction between HuR and *TRIM21* mRNA 3′UTR. RNA-immunoprecipitation using nonspecific and HuR specific antibodies from MCF7 cytoplasmic lysates revealed that the HuR protein was associated with *TRIM21* mRNA (Fig. 4A and Fig. S14). In addition, immunoprecipitation of UV-crosslinked and RNaseA-digested RNA–protein complexes from binding reactions of MCF7 cytoplasmic lysate, with radioactively labelled *TRIM21* 3′UTR RNA, showed binding of endogenous HuR to the *TRIM21* 3′UTR RNA (Fig. 4B and Fig. S15). RNA–protein UV-crosslinking assay showed dose-dependent interaction of purified HuR with radioactively labelled *TRIM21* 3′UTR RNA in vitro (Fig. 4C,D and Fig. S15). UV-crosslinking competition assay using radiolabelled *TRIM21* 3′UTR with three increasing concentrations of unlabelled *TRIM21* 3′UTR also demonstrated that the interaction between HuR and *TRIM21* 3′UTR is specific (Fig. 4C,D). Furthermore, UV-crosslinking assay with wild type and HuR binding-site mutant *TRIM21* 3′UTRs showed dose-dependent increase in the binding of purified HuR with the wild type *TRIM21* 3′UTR, whereas the binding was significantly reduced in case of mutant *TRIM21* 3′UTR (Fig. 4E and Fig. S15). Together, these findings suggested a specific interaction between RNA-binding protein HuR and *TRIM21* mRNA 3′UTR.

### Effect of HuR on *TRIM21* mRNA translation is mediated through its 3′UTR

**Figure 5:**
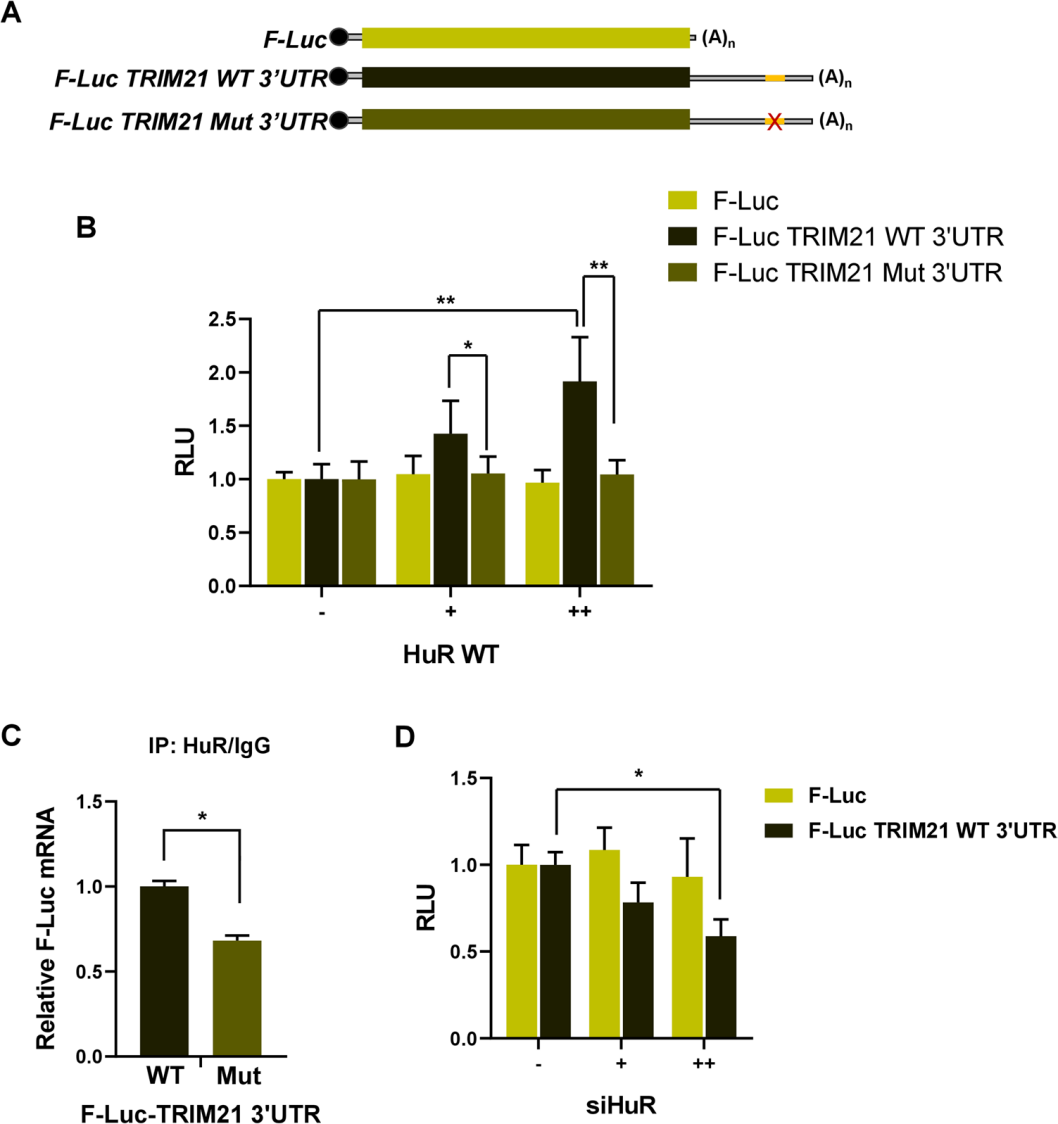
Effect of HuR on *TRIM21* mRNA translation is mediated through its 3′UTR. (**A**) Schematic representation of different *TRIM21 luciferase* reporter constructs. (**B**) Luciferase reporter assay was performed with the MCF7 cells transfected with different Firefly luciferase constructs, cotransfected with two increasing concentrations of WT Myc-tagged HuR expressing construct. (**C**) HuR and control IgG immunoprecipitation was performed with lysates of cells transfected with Firefly luciferase (having wild type or mutant *TRIM21* mRNA 3′UTR) reporter gene construct and a Renilla luciferase construct (as transfection control). qRT-PCR from the total RNA isolated from the immunoprecipitated samples was performed using F-Luc and R-Luc specific primers. The ratio of F-Luc/R-Luc RNA level was used to calculate fold change of HuR-immunoprecipitated RNA vs IgG-immunoprecipitated RNA in wild-type and mutant TRIM21 3′UTR transfected cells. (**D**) Luciferase reporter assay was performed with the MCF7 cells transfected with different Firefly luciferase constructs, cotransfected with two increasing concentrations of siRNA against endogenous *HuR*. F-Luc values are normalized to R-Luc values as transfection control. Data represents mean ± SD values from 3 independent experiments. *Signifies a *p* value ≤ 0.05, **signifies a *p* value ≤ 0.01 (paired two-tailed t-test).

To further understand the mechanism of HuR-mediated translation upregulation of TRIM21, luciferase assay with reporter constructs containing wild-type and HuR-binding site mutant *TRIM21* 3′UTRs was performed. MCF7 cells were cotransfected with different luciferase-*TRIM21* 3′UTR constructs and Myc-tagged HuR expressing construct (Fig. 5A). Overexpression of HuR showed a significant dose-dependent increase in luciferase activity from a reporter gene construct containing the wild type *TRIM21* 3′UTR, but not from a reporter gene containing the *TRIM21* 3′UTR with the mutant HuR binding site (Fig. 5B). Furthermore, immunoprecipitation of HuR showed significantly reduced interaction of HuR with the reporter mRNA containing mutant *TRIM21* 3′UTR mRNA compared to the wild type *TRIM21* 3′UTR mRNA (Fig. 5C and Fig. S7). Transfection of MCF7 cells with siRNA against *HuR* also showed a dose-dependent decrease in luciferase activity from the reporter gene construct containing the wild-type *TRIM21* 3′UTR (Fig. 5D). Collectively, these observations suggested that RNA-binding protein HuR increased the translation of *TRIM21* mRNA in a 3′UTR-dependent manner.

### Dihydrotanshinone-I prevents the upregulation of TRIM21 by HuR

**Figure 6:**
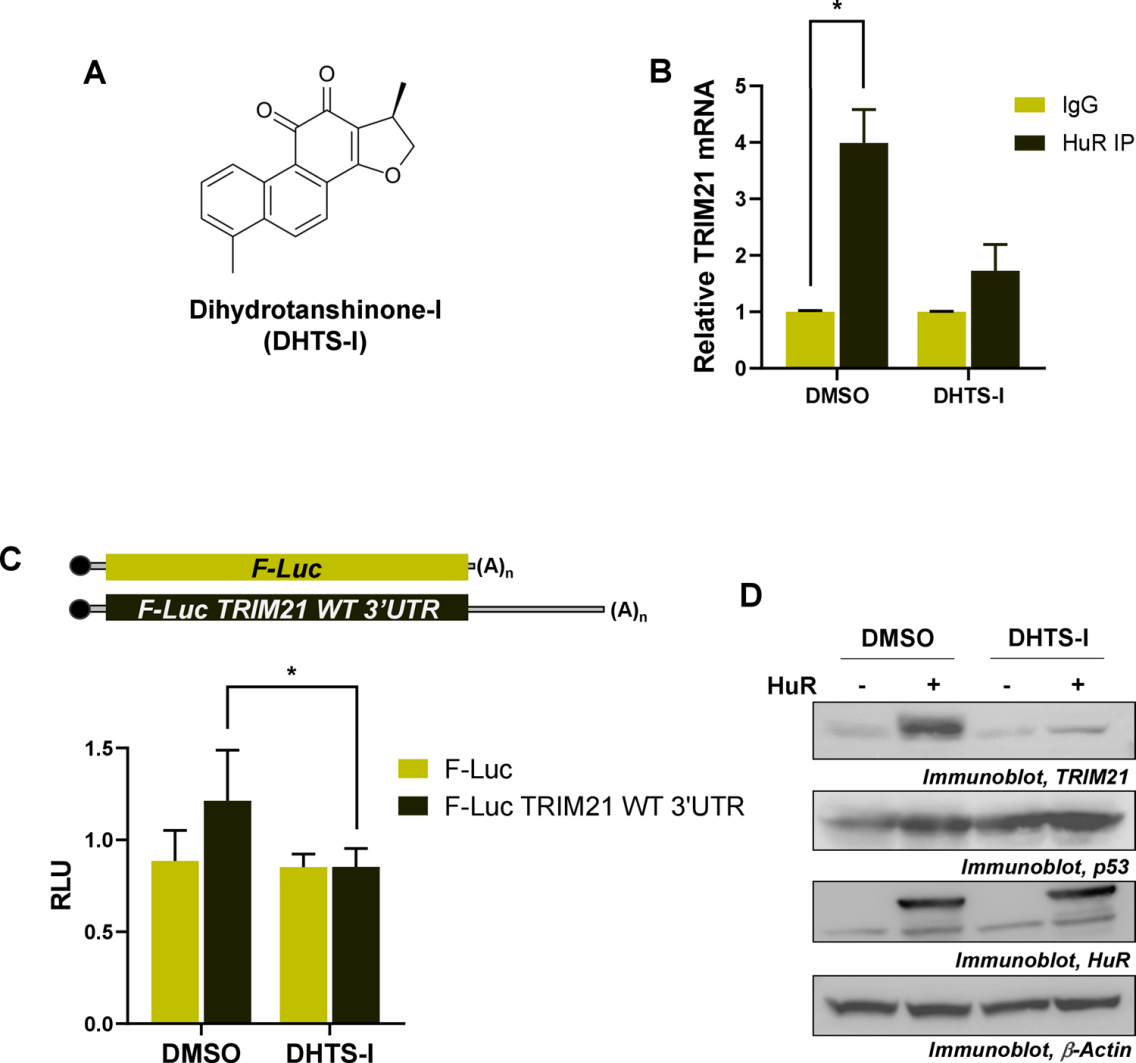
HuR-mediated translational upregulation of TRIM21 is prevented by DHTS-I. (**A**) Chemical Structure of dihydrotanshinone-I (DHTS-I). (**B**) HuR was RNA-immunoprecipitated from the lysates of the MCF7 cells treated or untreated with DHTS-I. qRT-PCR from the total RNA isolated from the immunoprecipitates was carried out using TRIM21 and GAPDH primers. (**C**) Luciferase reporter assay was performed with the MCF7 cells transfected with different Firefly luciferase constructs, treated with or without DHTS-I. F-Luc values are normalized to R-Luc values as transfection control. Data represents mean ± SD values from 3 independent experiments. *Signifies a *p* value ≤ 0.05, (paired one-tailed t-test). (**D**) MCF7 cells transfected with Myc-tagged HuR expressing construct, treated with or without DHTS-I were lysed and immunoblotted using TRIM21, p53, HuR and GAPDH antibodies.

Dihydrotanshinone-I (DHTS-I) is a natural product extracted from *Salvia miltiorrhiza*, and has been reported to potentially act as an anti-cancer drug (Fig. 6A). Evidence has suggested that DHTS-I increases the binding of HuR to the mRNAs with longer 3′UTR and with higher density of U/AU-rich elements. However, DHTS-I has also been shown to reduce the interaction of HuR with the mRNAs having shorter 3′UTR and with low density of U/AU-rich elements^38^. The *TRIM21* 3′UTR was found to have only one U-rich element where HuR was shown to bind. Therefore, we sought to check the effect of DHTS-I treatment on the HuR mediated translation upregulation of *TRIM21*. RNA-immunoprecipitation of HuR from DHTS-I treated and untreated MCF7 cells showed that the DHTS-I treatment reduced the interaction of HuR with *TRIM21* mRNA (Fig. 6B). Immunoprecipitation of HuR from UV treated and untreated MCF7 cells showed UV-irradiation increased interaction of HuR with *TRIM21* mRNA. However, the interaction was reduced when UV-irradiated MCF7 cells was treated with DHTS-I (Fig. S8A and Fig. S16). Similarly, siRNA-mediated knockdown of HuR also prevented the upregulation of TRIM21 level post UV-irradiation (Fig. S8B and Fig. S17). DHTS-I treatment also decreased the luciferase activity from the reporter gene construct containing the wild-type *TRIM21* 3′UTR (Fig. 6C). Finally, DHTS-I treatment was found to prevent the upregulation of TRIM21 by HuR. On the other hand, DHTS-I was found to enhance HuR-mediated upregulation of p53 protein (Fig. 6D and Fig. S18), which is likely due to the greater length and presence of high density of U/AU-rich elements in the 3′UTR of *p53* mRNA (Fig. S9).

## Discussion

E3 ubiquitin ligase TRIM21 is a posttranslational modifier which is primarily involved in immune functions. It is mainly involved in viral neutralization, cytokine production and inflammation regulation^5^. However, evidences have revealed that the TRIM21 is also associated with a large number of human cancers. It is found to decrease proliferation, colony formation and migration of hepatocellular carcinoma cells^23^. It also degrades snail, thereby preventing the epithelial to mesenchymal transition in breast cancer cells, one of the hallmarks of cancer^24,39^. Moreover, TRIM21 degrades anti-apoptotic molecule, Bcl2, thereby triggering apoptosis in cervical cancer cells^40^. Therefore, TRIM21 acts as tumor suppressor in most cancers. However, it also plays a pro-oncogenic role in certain cancers. For example, TRIM21 is involved in the proliferation of osteosarcoma cells^25^. It has also been shown to increase resistance of colon cancer to cisplatin by downregulating Par-4^41^. TRIM21 has also been reported to enhance cytoplasmic retention of guanosine 5′-monophosphate synthase (GMPS), thereby triggering Mdm2-mediated degradation of p53 under unstressed conditions^27^. However, the interaction between TRIM21 and GMPS could not be detected following genotoxic stress, thereby leading to the stabilization of p53^27^. Very recently, TRIM21 has been shown to degrade GMPS in collaboration with SERPINB5, thereby reducing p53 expression in nasopharyngeal cells (NPCs), which eventually leads to radio-resistance of NPC cells^28^. However, the function of TRIM21 under genotoxic condition has not been well elucidated. We have demonstrated that TRIM21-mediated proteasomal degradation in collaboration with miR-125b-mediated translation repression of HuR contribute to pulsatile change in p53 level following DNA damage^26^. In addition, TRIM21 knockdown was found to reduce number of viable cells post UV exposure^26^. Therefore, it is necessary to understand in detail the physiological role of TRIM21 in response to DNA damage.

**Figure 7:**
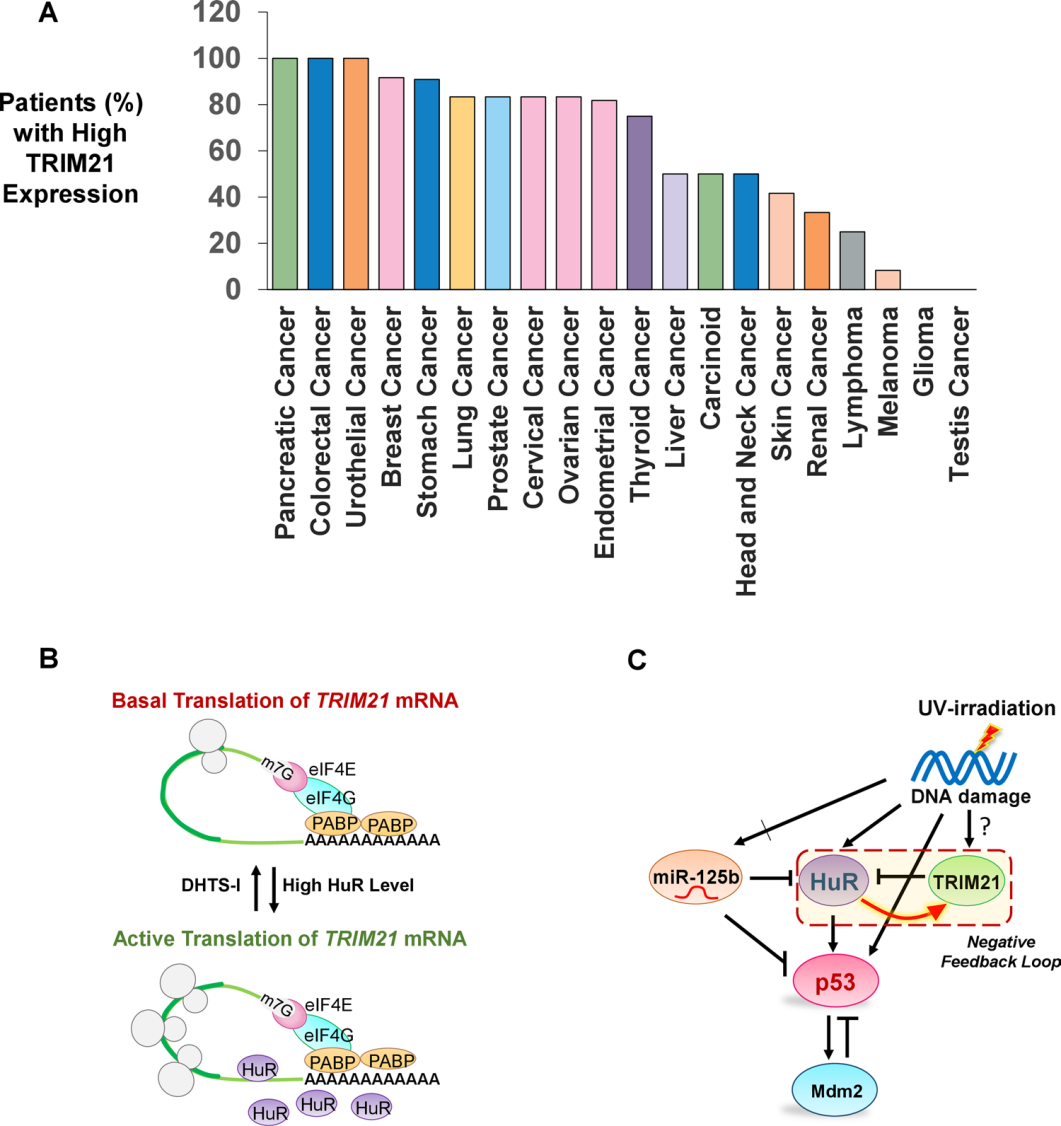
Model of HuR-mediated translation regulation of pro-oncogenic factor TRIM21 influencing the expression of p53 under genotoxic stress. (**A**) Plot of % of patients with different cancers with high level of TRIM21 expression^42^ (Protein Expression Summary from Human Protein Atlas, v19.proteinatlas.org/ENSG00000132109-TRIM21/pathology). (**B**) Proposed model of HuR-mediated translation activation of TRIM21. HuR overexpression enhances the rate of translation of *TRIM21* mRNA which can be reversed by DHTS-I treatment. (**C**) Network diagram representing the regulation of p53 mediated by HuR, miR-125b, TRIM21 and Mdm2 in response to UV-induced DNA damage, indicating the negative feedback loop between TRIM21 and HuR.

Previous reports have suggested that the depletion of TRIM21 in breast cancer cells is correlated with increased cell proliferation and tumor formation^30^. However, we found TRIM21 overexpression enhances proliferation as well as colony formation by breast cancer cells. Interestingly, TRIM21 overexpression reverses the decrease in cell proliferation and colony formation caused by HuR overexpression or UV treatment, thereby demonstrating a pro-oncogenic function of TRIM21. TRIM21 is also found to be upregulated in many cancer patients (Protein Expression Summary from Human Protein Atlas, v19.proteinatlas.org/ENSG00000132109-TRIM21/pathology) which further substantiates our observations about the pro-oncogenic function of TRIM21 (Fig. 7A)^42^. Therefore, it is of interest that a regulator such as TRIM21 has been discovered as a new component of the extensively studied DNA damage response pathway, providing new targets for therapeutic intervention in cancer.

TRIM21 is differentially expressed in multiple autoimmune diseases and cancers^5,23,41,43^. However, the detailed regulation of TRIM21 expression has never been well elucidated. TRIM21 has been shown to be regulated by three major mechanisms. Transcription of *TRIM21* is regulated by IRFs in response to IFN-α and IFN-β stimulations^29^. It is also regulated at mRNA level: for instance, miR-494-3p negatively affects the stability of *TRIM21* mRNA, thereby controlling the expression of TRIM21 protein^30^. LPS treatment has been shown to mediate mono-ubiquitination of TRIM21 followed by lysosomal degradation^44^. However, the translation regulation of TRIM21 has not been investigated. We have demonstrated for the first time HuR-mediated translation regulation of *TRIM21* mRNA. HuR is a ubiquitously expressed RNA-binding proteins which is a pivotal player in posttranscriptional regulation in response to stress conditions^45,46^. Photoactivatable Ribonucleoside Enhanced Crosslinking and Immunoprecipitation (PAR-CLIP) sequencing has revealed ~ 1700 mRNAs which have HuR-binding sites in their 3′UTR^33,34^. These HuR targets are translated to proteins which are involved in cell proliferation, differentiation and apoptosis^46^. HuR has also been associated with different diseases, including chronic inflammation, cardiovascular disease and cancers^45^. HuR is predominantly nuclear and has been thought to be involved in mRNA splicing^47^. However, upon stress stimulation, it translocates from nucleus to cytoplasm and enhances the stability and translation of AU/U-rich elements containing mRNAs. HuR often acts by antagonizing the action of microRNAs which are the negative regulators of mRNA stability and translation^49,31,48^. Nevertheless, evidence have been put forward to show the direct action of HuR on mRNA stability as well as translation^50^. We found HuR interacts with the U-rich element in the 3′UTR of *TRIM21* and triggers the translation activation of *TRIM21* mRNA. However, mutation in the HuR binding site in the 3′UTR of *TRIM21* abrogates the binding, thereby affecting the translation activation. Furthermore, DHTS-I treatment reduces the interaction of HuR with the *TRIM21* mRNA, thereby repressing the translation of *TRIM21*. Interestingly, we found DHTS-I treatment decreases the HuR-mediated upregulation of TRIM21, whereas it further increases the HuR-mediated upregulation of p53, thereby supporting its role as a prospective anticancer agent.

Therefore, based on our observations, we propose a model in which increase in cytoplasmic HuR level increases the binding of HuR to the U-rich binding site in the *TRIM21* 3′UTR, which subsequently leads to translational activation of TRIM21. On the other hand, DHTS-I treatment reduces the binding of HuR to the *TRIM21* 3′UTR, thereby allowing basal level translation (Fig. 7B). Thus, an increased HuR level enhances TRIM21 expression, which in turn reduces HuR to a low steady state level by inducing protein degradation, thereby constituting a negative feedback loop under UV irradiation. Therefore, this negative feedback loop may add a new layer of regulation in the p53 control network activated upon UV irradiation (Fig. 7C). Negative feedback loops reduce response time to stimuli and achieve steady state concentrations of proteins faster, allowing faster and more fine-tuned response to stimuli^51^. The HuR-TRIM21 negative feedback loop may allow the level of HuR, a highly stable protein, to achieve a low steady state faster in response to genotoxic stress, which is required for the pulsatile expression of p53. Moreover, the negative feedback loop gives rise to inversely proportional steady state concentrations of TRIM21 and HuR in cells, which may be the reason for both TRIM21 and HuR to show either oncogenic or tumor-suppressive roles in the context of different cancers, depending upon their relative concentrations. Future studies are warranted to understand this intricate control system under DNA damage condition in order to elucidate the role of TRIM21 in tumorigenesis. In addition, since DHTS-I differentially controls the HuR-mediated expression of TRIM21 (a pro-oncogenic factor) and p53 (an anti-oncogenic factor), it has the potential of being developed as a therapeutic to treat autoimmune disorders and cancers.

## Methods

### Plasmid constructs

The 357 nucleotides *TRIM21* 3′UTR containing the putative U-rich HuR binding site (GUUUUUUUUUU) was amplified from human monocytic (U937) RNA by RT-PCR and cloned in pCDNA3.1 vector or cloned downstream of firefly luciferase gene in pCDNA3-Fluc vector. The predicted HuR binding site was mutated (GUUUUUUUUUU → CGCGCACGCCA) using mega primer-based site directed mutagenesis and cloned into the same vector. TRIM21, HuR WT and HuR K182R plasmid constructs were used for overexpression as described earlier^26^. pRL-CMV and pGEM-T Easy plasmid constructs were used for transfection normalization.

### Cell culture, treatment and transfection

MCF7 and MDA-MB-231 human breast carcinoma cells were grown in 0.22 μm filter (Millipore) sterilized Dulbecco’s modified Eagle’s Medium–High Glucose (Thermo Fisher Scientific) pH 7.4, supplemented with 10% FBS (Thermo Fisher Scientific) and 1% PEN-STREP (Thermo Fisher Scientific) at 37˚C in a 5% CO_2_ incubator. Cells were exposed to 10 J/m^2 ^pulse of UVC irradiation in UV crosslinker (Strata linker). Cells were treated with 100 µg/mL cycloheximide (Amresco) or 10 µM dihydrotanshinone-I (Sigma Aldrich). Cells were transfected with plasmid vectors described above or siRNAs (siGENOME SMART pools, HuR or TRIM21) and Non-Targeting siRNA pool or control oligo (Dharmacon) using Lipofectamine 2000 (Thermo Fisher Scientific) or TurboFect (Thermo Fisher Scientific) in serum-free OptiMEM (Thermo Fisher Scientific) or Low Glucose DMEM media (Thermo Fisher Scientific).

### Cell lysate preparation

Cells were harvested at different time points post treatment or transfection, pelleted down and washed with 1 × PBS. The cell pellet was resuspended in S10-lysis buffer (10 mM HEPES pH-7.4, 15 mM KCl, 1 mM PMSF, 1 mM DTT, and 0.1% Triton X100)^31^ or NT2 buffer (50 mM Tris–HCl pH 7.4, 150 mM NaCl, 1 mM MgCl_2_, 0.05% NP-40)^52^ or RIPA buffer (5 mM Tris–HCl pH 7.4, 150 mM NaCl, 1% Triton X100, 0.1% SDS, 1% sodium deoxycholate)^53^ or Polysome Lysis buffer (20 mM Tris–HCl/ 10 mM HEPES pH 7.4, 5 mM MgCl_2_, 150 mM NaCl/ 100 mM KCl, 1 mM DTT, 0.5% NP-40, 100 U/mL RNase inhibitor, 0.2 mM PMSF, 1X Protease Inhibitor Cocktail)^31,54^ and were passed through pipette for several times. These were then centrifuged at 10,000 r.p.m for 30 min and supernatant was collected for further experiments.

### Immunoblotting

Cell lysates were quantified using Bradford reagent (Amresco). Equal amount of protein was loaded and resolved using 10% SDS–Polyacrylamide Gel electrophoresis. Proteins were transferred to PVDF membrane (Millipore) at constant current (1 mA/cm^2^) using semi-dry transfer apparatus (TE70X, Hoefer). This was followed by immunoblotting using anti- HuR (3A2, Santacruz Biotechnology), p53 (DO-1, Santa Cruz Biotechnology), TRIM21 (E-11, Santa Cruz Biotechnology) or (D101D, Cell Signaling Technologies), Myc (71D10, Cell Signaling Technologies), GAPDH (FL-335, Santacruz Biotechnology), and β-Actin (A00730, Genscript) primary antibodies. HRP conjugated anti-Mouse (7076S) (Cell Signaling) and anti-Rabbit (7074S, Cell Signaling) were used as secondary antibodies. Bands were detected using Femtolucent ECL Plus detection kit (Geno Bioscience).

### RNA-immunoprecipitation

Untreated or DHTS-I treated MCF7 cells were lysed in S10 buffer (pH 7.4) supplemented with 1 mM ATP and 1 mM MgCl_2_. The lysate was quantified and pre-cleared for 30mins at 4 °C. Protein-A Sepharose beads (Sigma Aldrich) were coated for 10–16 h at 4 °C using Control IgG (Santacruz Biotechnology), and HuR (Santacruz Biotechnology) antibodies in NT2 buffer. 1 mg pre-cleared cell lysate was incubated with antibody-coated beads for 2hours at room temperature. Unbound proteins were removed by washing with NT2 buffer. RNA was isolated from the beads using RNAiso Plus (Takara) and cDNA was prepared using M-MLV Reverse Transcriptase (Thermo Fisher Scientific).

### RNA analysis

Total RNA was isolated by RNAiso Plus (Takara) reagents and cDNA was synthesized using oligo(dT) primer by M-MLV reverse transcriptase (Thermo Fisher). *TRIM21* 3′UTR-specific primers were used for detection of *TRIM21* mRNA, while *GAPDH* specific primers were used for mRNA normalisation. Semi-quantitative RT-PCR was done using TRIM21, GAPDH primers. Cycle numbers were determined in order to prevent the saturation of the amplicon level.

### In vitro RNA–protein UV crosslinking assay

^32^P-UTP labelled *TRIM21* RNAs (both wild type and mutant type) were in vitro transcribed from linearized plasmid DNA templates using T7 RNA polymerase (New England Biolabs). Radiolabelled RNAs of equal specific activity were UV-crosslinked with purified 6X-His-tagged HuR protein or cell lysate and the unbound RNAs were digested using RNase-A as described earlier^55^. The RNA protein complexes were then resolved on 12% SDS-PAGE gel and the gels were dried in gel drier (BioRad) for phosphorimaging using Typhoon Trio (GE Healthcare). UV-crosslinking competition assay was performed using purified HuR and increasing concentrations of unlabelled competitor RNAs before incubation with radiolabelled RNA. This was followed by UV-crosslinking, RNase digestion, gel electrophoresis and phosphorimaging.

### Polysome analysis

Transfected cells were incubated in cycloheximide for 30 min prior to lysate preparation. Cells were lysed in Polysome lysis buffer containing cycloheximide and the extract was loaded on a 10–50% sucrose gradient followed by centrifugation at 30,000 r.p.m for 4 h. Fractions were collected using a programmable gradient fractionator (Biocomp, Canada) and absorbance at 254 nm was measured^56^. RNA was isolated from all the fractions and detected by semi-quantitative PCR using *TRIM21*- and *GAPDH*-specific primers.

### Luciferase reporter assay

Cells were treated with DHTS-I or co-transfected with different *TRIM21* 3′UTR luciferase reporter constructs, HuR expressing plasmid construct or siRNA against endogenous *HuR*. Dual luciferase reporter assay (Promega) was performed post 48 h of transfection or post 4 h of treatment in Chameleon multimode plate reader (Hidex) as per manufacturer’s instructions.

### Cell proliferation and Colony formation assay

48 h post transfection MCF7 cells were treated with or without UVC. 4 h post treatment, 10^3^ MCF7 cells were seeded and cell proliferation was determined at 24, 48 and 72 h using the MTT (Sigma Aldrich) based cell growth determination kit. Transfected MCF7 cells, with or without UV exposure, were similarly seeded in 6 well plates and were allowed to grow for 2 weeks. Colonies were stained with crystal violet post two weeks of seeding and CFUs (colony forming units) were counted.

### In silico analysis

Human *TRIM21* 3′UTR mRNA sequence (357 nucleotides) was analysed using RBPDB (https://rbpdb.ccbr.utoronto.ca/) and RBPmap (https://rbpmap.technion.ac.il/) for predicting the HuR-binding site in the 3′UTR. In addition, mFold RNA structure predicting web server (https://unafold.rna.albany.edu/?q=mfold/RNA-Folding-Form) was used to predict the structure of the *TRIM21* 3′UTR RNA.

### Statistical analysis

All graphical data represent mean ± standard deviation of at least two biological replicates, each with two technical replicates. *Signifies a *p* value ≤ 0.05, **signifies a *p* value ≤ 0.01, ***signifies a *p* value ≤ 0.005 (Paired two-tailed or one-tailed Students t test as applicable) between controls and samples indicated in the figures.

## Supplementary information

Supplementary file1 (PDF 2146 kb)
