# Supplementary material for: Negative feedback regulation by HuR controls TRIM21 expression and function in response to UV radiation
Source: Sci Rep. 2020 Jul 16;10:11753. doi: 10.1038/s41598-020-68646-3 (PMC7367240; doi:10.1038/s41598-020-68646-3)
Supplement: Supplementary file 1 — Supplementary file1 (PDF 2146 kb) [file 41598_2020_68646_MOESM1_ESM.pdf]

Supplementary Information

Negative Feedback Regulation by HuR Controls TRIM21 Expression and Function in Response to UV Radiation

Abhishek Guha, Sharanya Nag and Partho Sarothi Ray*

Department of Biological Sciences, Indian Institute of Science Education and Research (IISER) Kolkata, Mohanpur 741246, West Bengal, India

* Correspondence: Tel: +91 9874703899 Email: psray@iiserkol.ac.in

Fig. S1

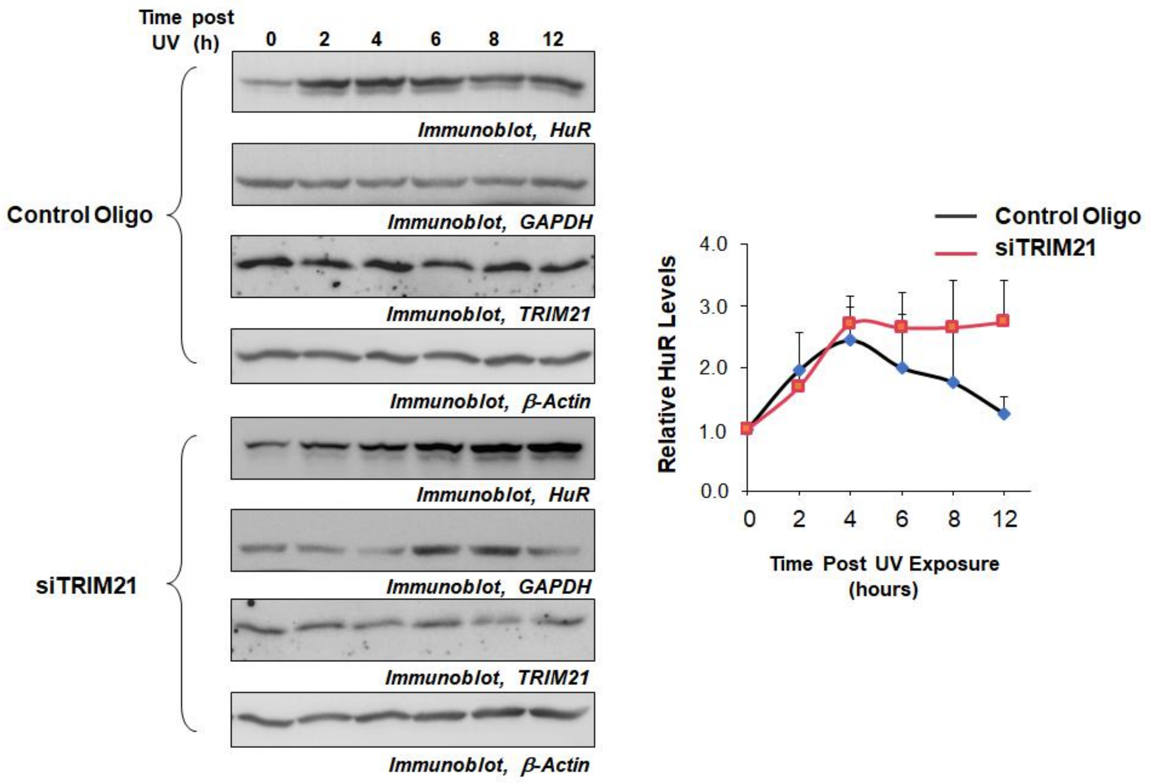

Supplementary Fig. 1. Cytoplasmic lysates of MCF7 cells transfected with control oligo or siTRIM21 and exposed to UV were collected at designated time points post UV exposure and immunoblotted with HuR, GAPDH, TRIM21 and β-Actin antibodies (left). Plot represents relative HuR band intensities from three independent experiments. See also Fig. S19 and S20.

Fig. S2

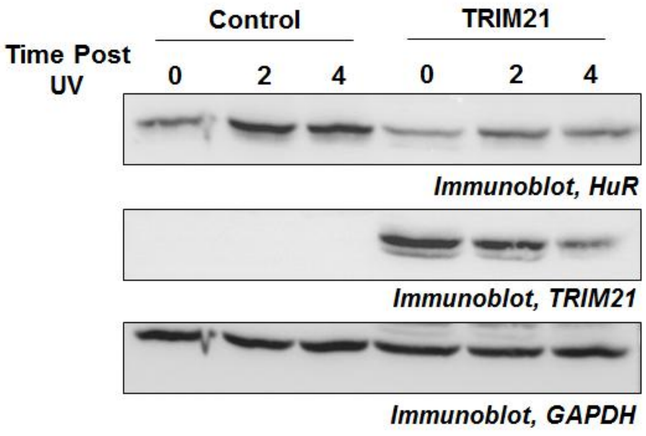

Supplementary Fig. 2. MCF7 cells transfected with empty vector (control) or TRIM21 expressing construct were exposed to UV. Cells were collected at designated time points post UV exposure, lysed, and the lysates were immunoblotted with HuR, TRIM21 and GAPDH antibodies. See also Fig. S21.

Fig. S3

HuR binding site in TRIM21 3'UTR from CLIP/PAR-CLIP data (Ref. 32)

TGGCTTTCTCTGGACACTGCCACTCTCCCCATTGGCACCGCTTCTCAGCCACAAACCCTGCCTCTTTTCCCCATG
AACTCTGAACCACCTTTGTCTCTGCAGAGGCATCCGGATCCCAGCAAGCGAGCTTTAGCAGGGAAGTCACTTC
ACCATCAACATTCCTGCCCCAGATGGCTTTGTGATTCCCTCCAGTGAAGCAGCCTCCTTATATTTGGCCCAAACT
CATCTTGATCAACCAAAAACATGTTTCTGCCTTCTT**TATGGGACTTAAGTTTTTTTTTTCTCCTCTCCATCTCTAG**
GATGTCGTCTTTGGTGAGATCTCTATTATATCTTGTATGGTTTGCAAAAGGGCTTCCT

HuR binding site in TRIM21 3'UTR from PAR-CLIP data (Ref. 33)

TGGCTTTCTCTGGACACTGCCACTCTCCCCATTGGCACCGCTTCTCAGCCACAAACCCTGCCTCTTTTCCCCATG
AACTCTGAACCACCTTTGTCTCTGCAGAGGCATCCGGATCCCAGCAAGCGAGCTTTAGCAGGGAAGTCACTTC
ACCATCAACATTCCTGCCCCAGATGGCTTTGTGATTCCCTCCAGTGAAGCAGCCTCCTTATATTTGGCCCAAACT
CATCTTGATCAACCAAAAACATGTTTCTGCCTTCTTTATGGGACTTAAGT**TTTTTTTTTCTCCTCTCCATCTCTAG**
GATGTCGTCTTTGGTGAGATCTCTATTATATCTTGTATGGTTTGCAAAAGGGCTTCCT

HuR binding site in TRIM21 3'UTR from PAR-CLIP data (Ref. 34,35)

TGGCTTTCTCTGGACACTGCCACTCTCCCCATTGGCACCGCTTCTCAGCCACAAACCCTGCCTCTTTTCCCCATG
AACTCTGAACCACCTTTGTCTCTGCAGAGGCATCCGGATCCCAGCAAGCGAGCTTTAGCAGGGAAGTCACTTC
ACCATCAACATTCCTGCCCCAGATGGCTTTGTGATTCCCTCCAGTGAAGCAGCCTCCTTATATTTGGCCCAAACT
CATCTTGATCAACCAAAAACATGTTTCTGCCTTCTTTATGGGACTTAAG**TTTTTTTTTTCTCCTCTCCATCTCTAG**
GATGTCGTCTTTGGTGAGATCTCTATTATATCTTGTATGGTTTGCAAAAGGGCTTCCT

HuR binding site from prediction algorithms (Ref. 36,37)

TGGCTTTCTCTGGACACTGCCACTCTCCCCATTGGCACCGCTTCTCAGCCACAAACCCTGCCTCTTTTCCCCATG
AACTCTGAACCACCTTTGTCTCTGCAGAGGCATCCGGATCCCAGCAAGCGAGCTTTAGCAGGGAAGTCACTTC
ACCATCAACATTCCTGCCCCAGATGGCTTTGTGATTCCCTCCAGTGAAGCAGCCTCCTTATATTTGGCCCAAACT
CATCTTGATCAACCAAAAACATGTTTCTGCCTTCTTTATGGGACTTAA**GTTTTTTTTTT**CTCCTCTCCATCTCTAG
GATGTCGTCTTTGGTGAGATCTCTATTATATCTTGTATGGTTTGCAAAAGGGCTTCCT

Supplementary Fig. 3. *TRIM21* mRNA 3'UTR sequence showing HuR binding region from three PAR-CLIP/CLIP studies (in red) and from predicted HuR binding-site within this region (in green).

Fig. S4

RBPDB Sequence Scan Results

Predictions for sequence: *TRIM21 3'UTR*

Score	Relative Score	RBP Name	Start	End	Matching sequence
4.40359056	100%	ELAVL1	339	342	GUUU
**4.40359056**	**100%**	**ELAVL1**	**272**	**275**	**GUUU**
4.40359056	100%	ELAVL1	246	249	GUUU
3.82636396	86%	ELAVL1	210	213	AUUU

http://rbpdb.ccbr.utoronto.ca/

RBPmap Prediction Results

Predictions for sequence: *TRIM21 3'UTR*
Genome: Human (hg38)
Selected motifs: HuR (Hs/Mm): *UU(G/U)(A/G)RUUU*
Stringency level: High
Conservation filter: Off
= =
Protein: HuR (Hs/Mm)

Position	Motif	Occurrence	Z-score	P-value
273	*UUKRUUU*	*GGACUUAAGUUUUUUUUUUCUCCUC*	3.286	5.08e-04
274	*UUKRUUU*	*GACUUAAGUUUUUUUUUUCUCCUCU*	3.286	5.08e-04
275	*UUKRUUU*	*ACUUAAGUUUUUUUUUUCUCCUCUC*	3.286	5.08e-04
276	*UUKRUUU*	*CUUAAGUUUUUUUUUUCUCCUCUCC*	3.286	5.08e-04

http://rbpmap.technion.ac.il/

Supplementary Fig. 4. Prediction analysis for HuR binding site in the *TRIM21* mRNA 3'UTR using RNA-binding protein database and RBPmap.

Fig. S5

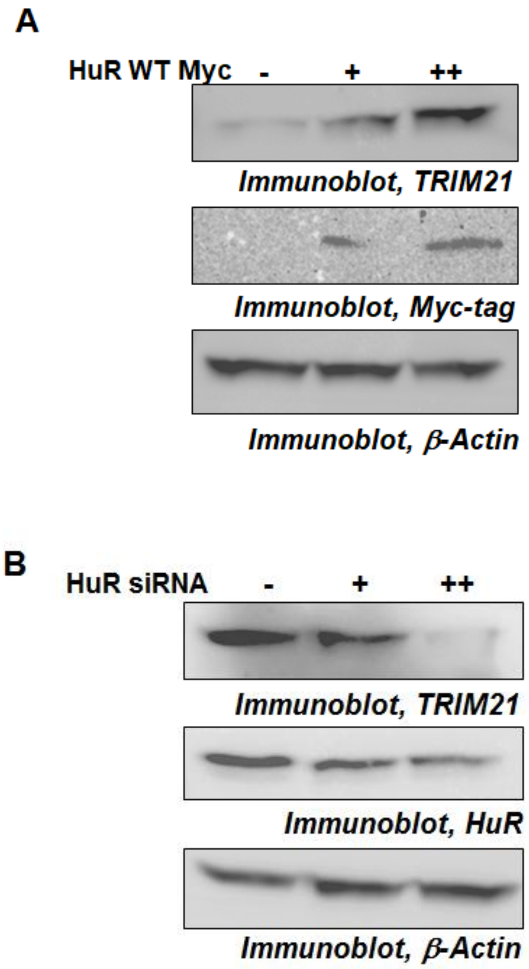

Supplementary Fig. 5. (A) Immunoblots of lysates of MDA-MB-231 cells transfected with two increasing concentrations of Myc-tagged-HuR (WT) expressing constructs probed with TRIM21, Myc-tag, and β-Actin antibodies. (B) Immunoblots of lysates of MDA-MB-231 cells transfected with two increasing concentrations of siRNA against endogenous *HuR* probed with TRIM21, HuR, and β-Actin antibodies. See also Fig. S22.

Fig. S6

$$Translational\ Index = \frac{Protein\ Levels}{Corresponding\ mRNA\ Levels}$$

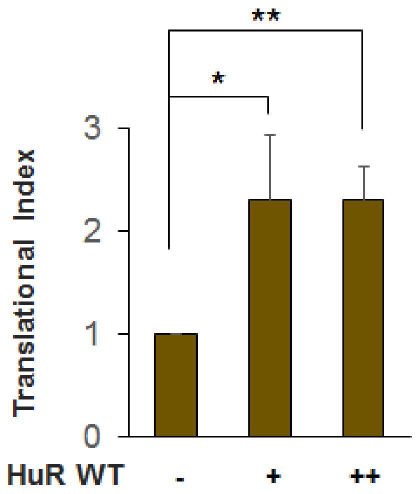

Supplementary Fig. 6. Translational index calculated from three independent experiments as described in Fig. 2C. Data represents mean ± SD values from 3 independent experiments. * signifies a P-value⩽0.05, ** signifies a P-value⩽0.01 (paired two-tailed t-test).

Fig. S7

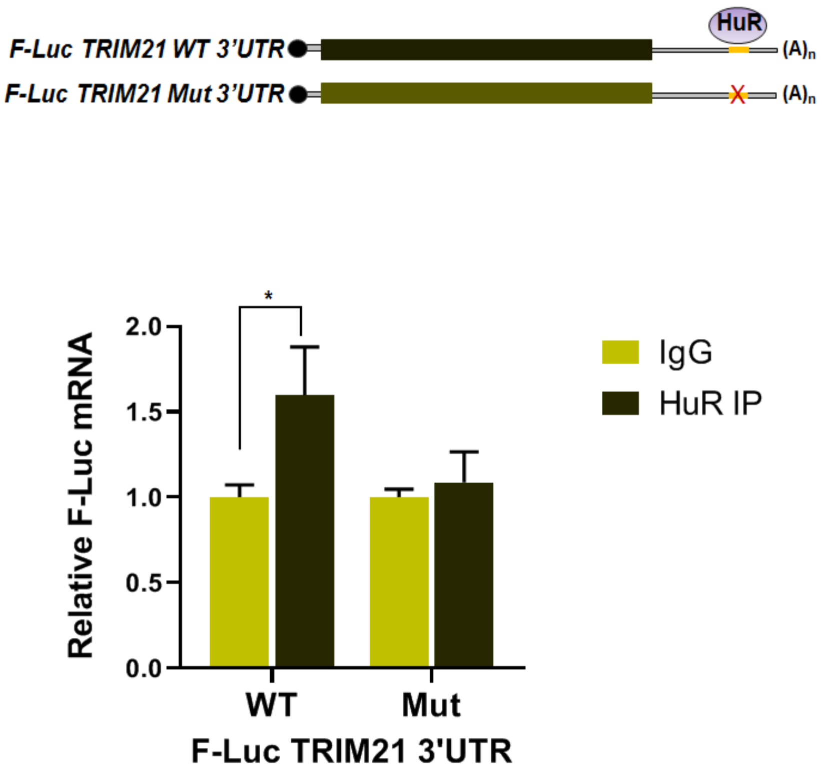

Supplementary Fig. 7. Detailed analysis of HuR RNA-immunoprecipitation from cells transfected with F-Luc-*TRIM21* WT 3'UTR and F-Luc-*TRIM21* MT 3'UTR shown in Fig. 5C. The mRNA levels are shown as fold change over that from IgG immunoprecipitation. Data represents mean ± SD values from 3 independent experiments. * signifies a P-value⩽0.05 (paired one-tailed t-test).

Fig. S8

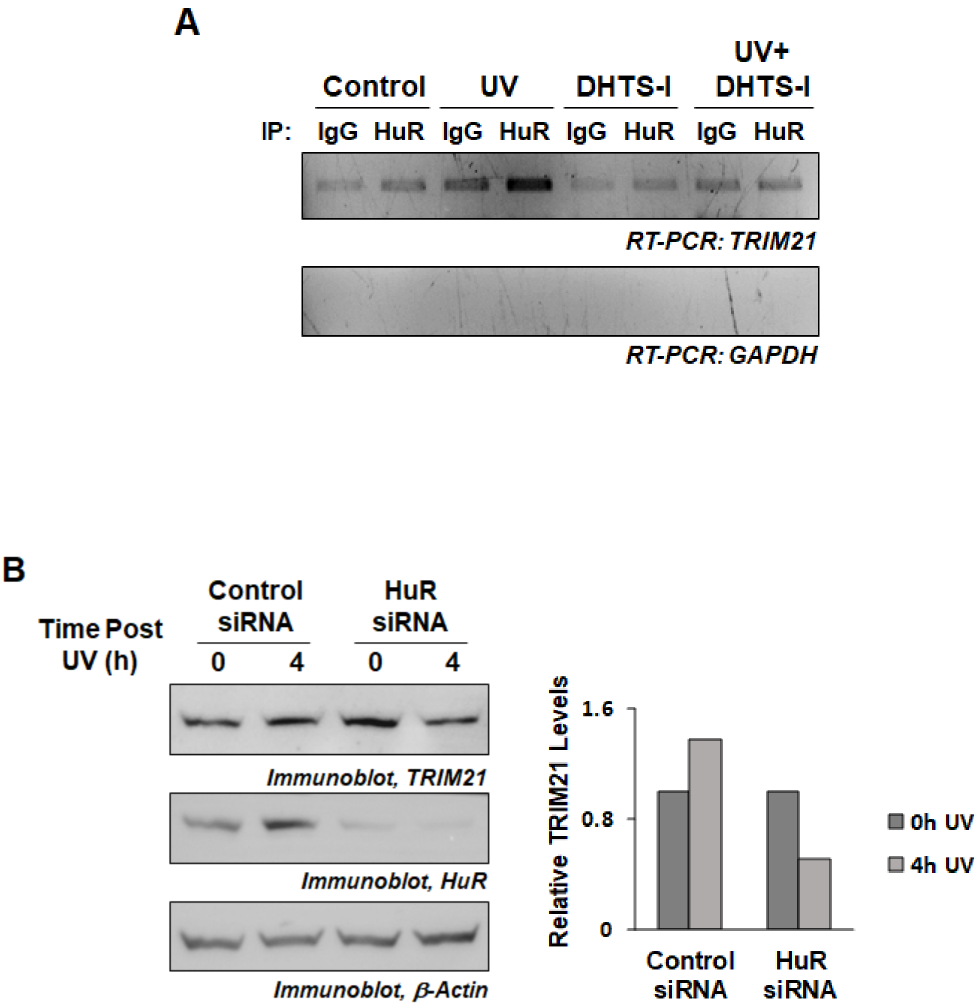

Supplementary Fig. 8. (A) HuR was immunoprecipitated from the lysates of the MCF7 cells treated with UV, DHTS-I or both or kept untreated. qRT-PCR from the total RNA isolated from the immunoprecipitates was carried out using TRIM21- and GAPDH-specific primers. (B) Immunoblotting from the lysates of control siRNA and HuR siRNA-treated samples post 4 hours of UV exposure using TRIM21, HuR and β-Actin antibodies. See also Fig. S15 and S16.

Fig. S9

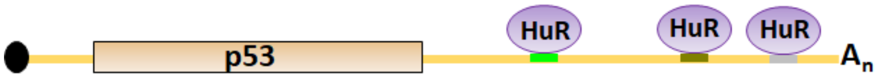

p53 3’UTR **(Partial Sequence with HuR Binding Sites)**

5’AACUUGACCCCCUUGAGGGUGCUUGUUCCCUCUCCCUGUUGGUCGGUGGGUUGGUAGUUUCUACAGU
UGGGCAGCUGGUUAGGUAGAGGGAGUUGUCAAGUCUCUGCUGGCCCAGCCAAACCCUGUCUGACAACC
UCUUGGUGAACCUUAGUACCUAAAAGGAAAUCUCACCCCAUCCCACACCCUGGAGGAUUUCAUCUCUUG
UAUAUGAUGAUCUGGAUCCACCAAGACUUGUUUUAUGCUCAGGGU**CAAUUUCUUUUUCUUUUUUUUUU**
UUUUUUUUCUUUUUCUUUGAGACUGGGUCUCGCUUUGUUGCCCAGGCUGGAGUGGAGUGGCGUGAUCU
UGGCUUACUGCAGCCUUUGCCUCCCCGGCUCGAGCAGUCCUGCCUCAGCCUCCGGAGUAGCUGGGACC
ACAGGUUCAUGCCACCAUGGCCAGCCAACUUUUGCAUGUUUUGUAGAGAUGGGGUCUCACAGUGUUGC
CCAGGCUGGUCUCAAACUCCUGGGCUCAGGCGAUCCACCUGUCUCAGCCUCCCAGAGUGCUGGGAUUA
CAAUUGUGAGCCACCACGUCCAGCUGGAAGGG**UCAACAUCUUUUACAUUCUG**CAAGCACAUCUGCAUUU
UCACCCCACCCUUCCCCUCCUUCUCCC**UUUUUAUAUCCCAUUUUUAUAUCGAUCUCUUAUUUUACAAUA**
AAACUUUGCUGCCACCUGUGUGUCUGAGGGGUG3’

Proximal HuR Binding Site

Middle HuR Binding Site

Distal HuR Binding Site

Supplementary Fig. 9. Sequence of *p53 3′UTR* (partial sequence) showing three HuR binding sites[31].

Fig. S10

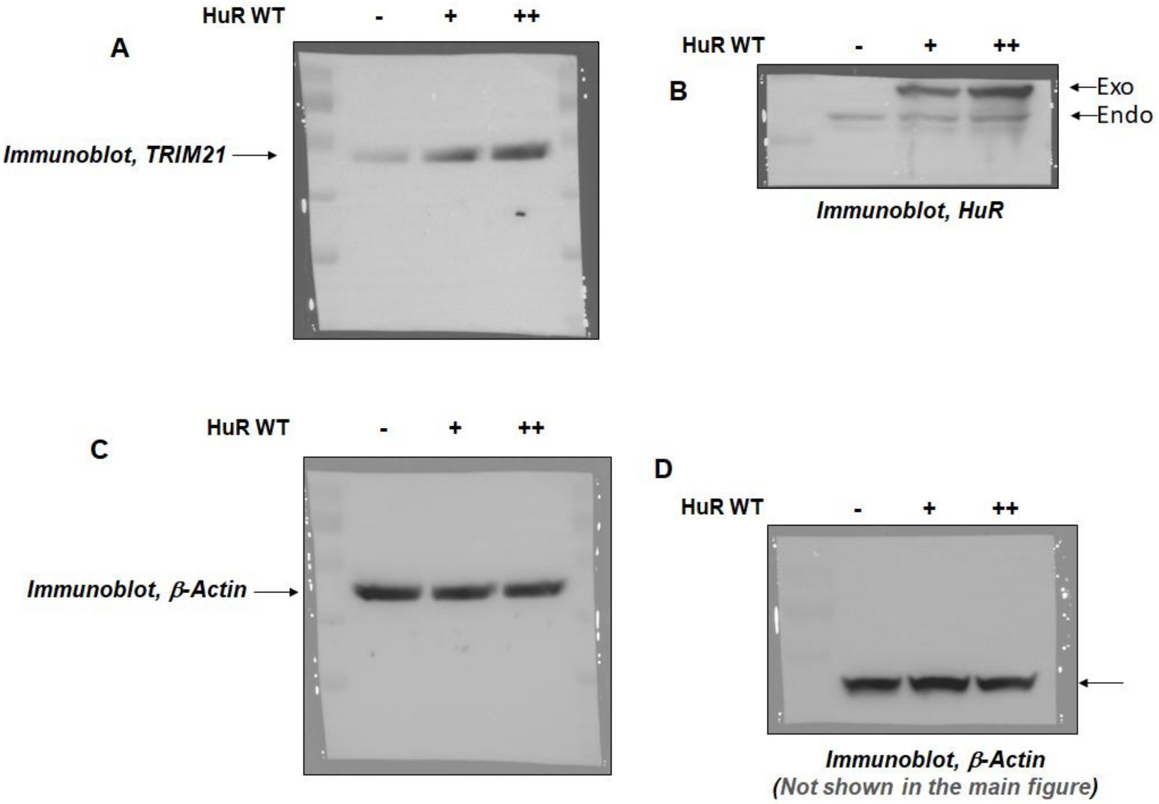

Supplementary Fig. 10. Full length blots (uncropped) of (A) TRIM21, (B) HuR and (C and D) β-Actin shown in Fig. 2C.

Fig. S11

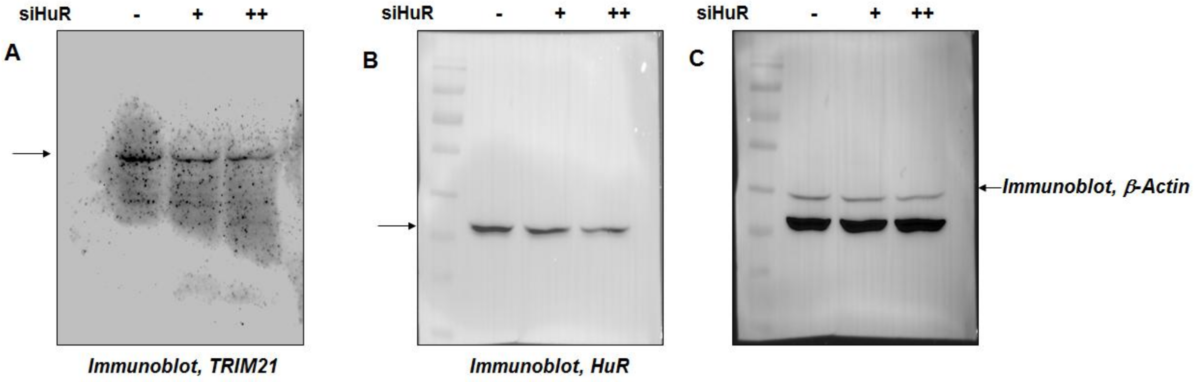

Supplementary Fig. 11. Full length blots (uncropped) of (A) TRIM21, (B) HuR, and (C) β-Actin shown in Fig. 2D. The lower band in (C) is of HuR in the unstripped blot reprobed with β-Actin antibody.

Fig. S12

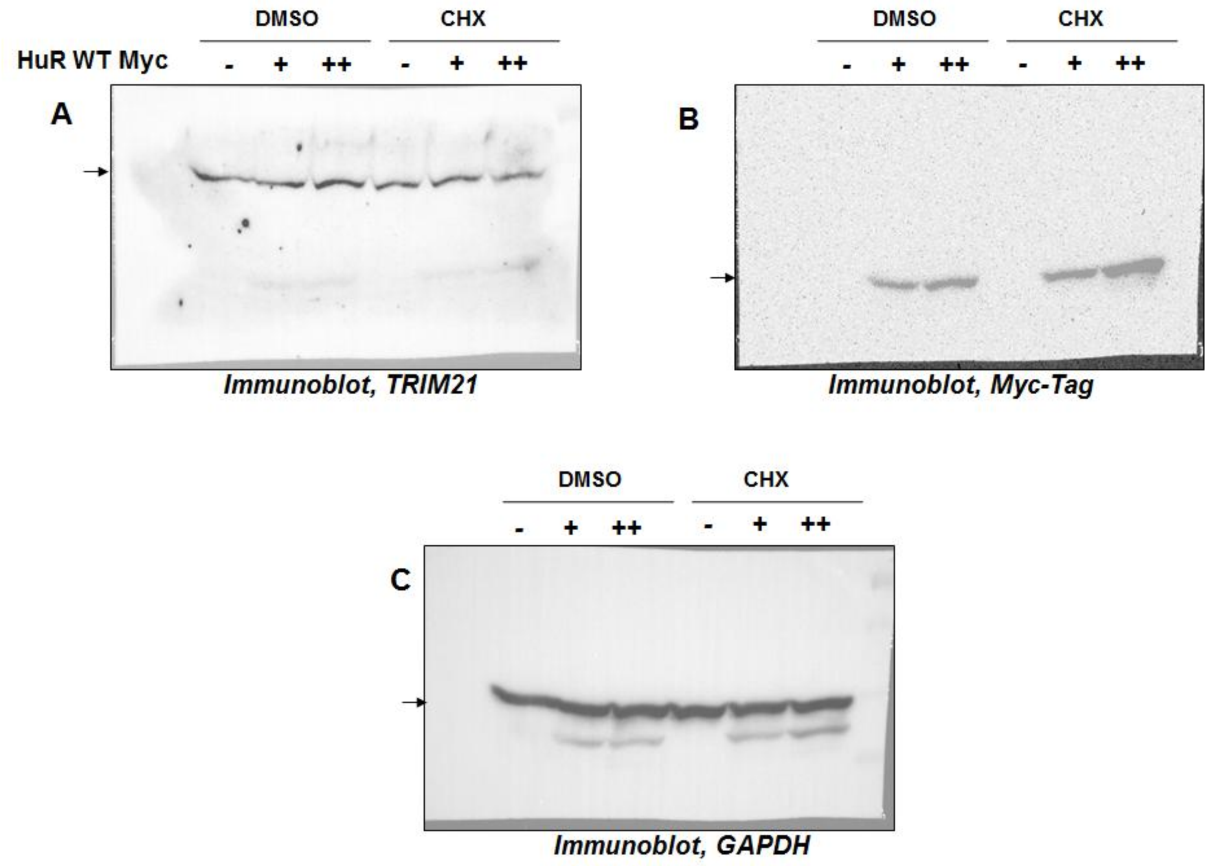

Supplementary Fig. 12. Full length blots (uncropped) of (A) TRIM21, (B) Myc-tagged HuR and (C) β-Actin shown in Fig. 3A. The lower band in (C) is of Myc-tagged HuR in the unstripped blot reprobed with GAPDH antibody.

Fig. S13

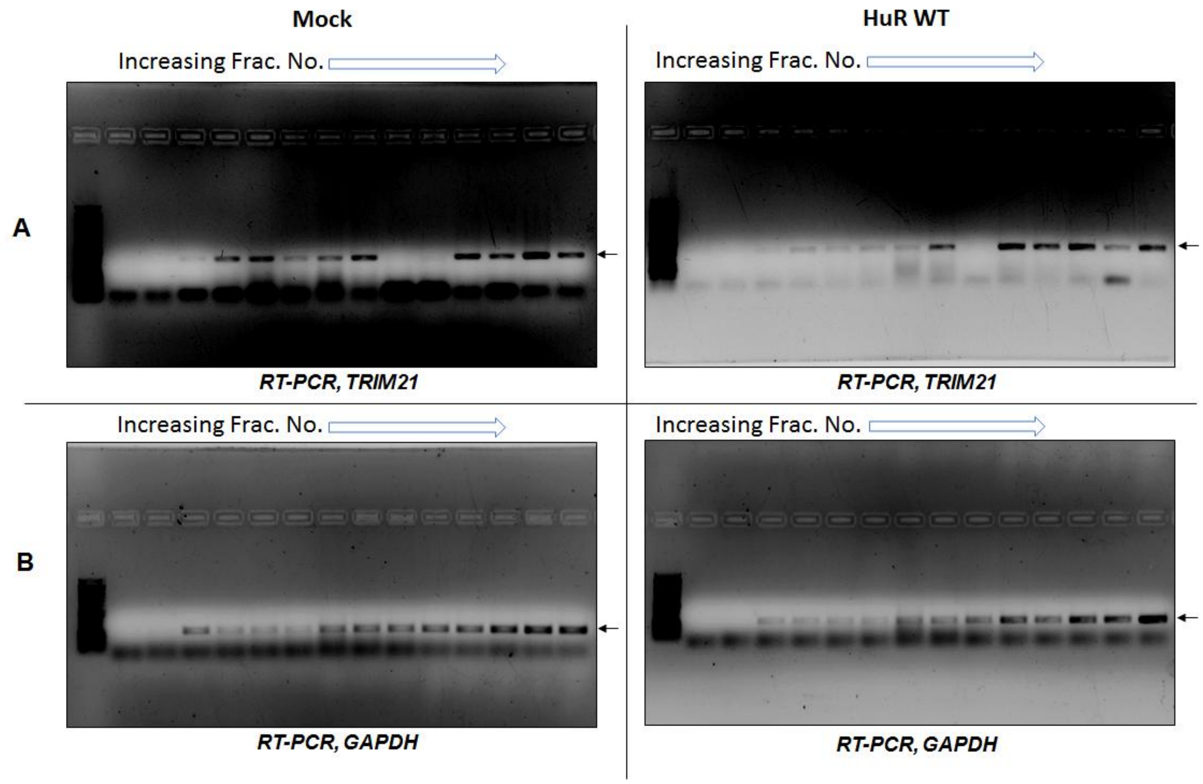

Supplementary Fig. 13. Full length ethidium bromide stained agarose gel (uncropped) of (A) *TRIM21* and (B) *GAPDH* shown in Fig. 3B.

Fig. S14

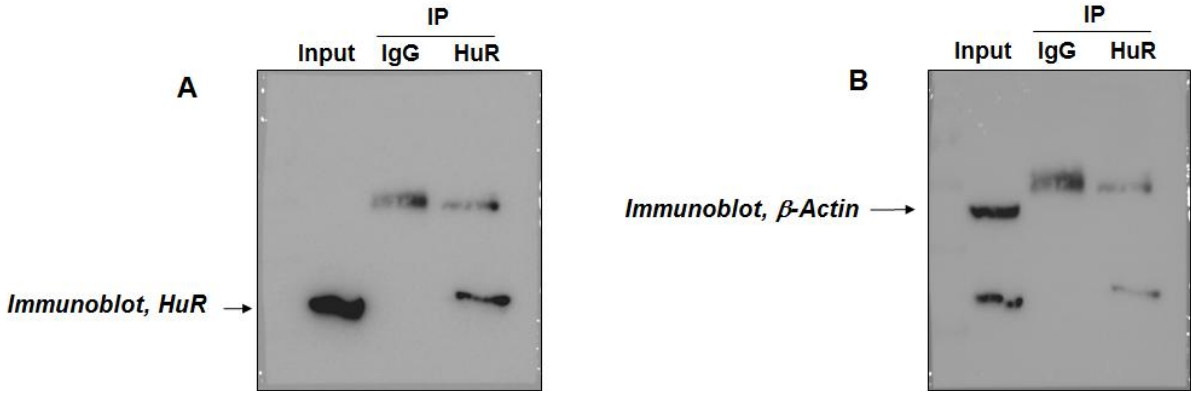

Supplementary Fig. 14. Full length blots (uncropped) of (A) HuR, and (B) β-Actin shown in Fig. 4A. The lower band in (B) is of HuR in the unstripped blot reprobed with Actin antibody and upper bands are of IgG Heavy Chain.

Fig. S15

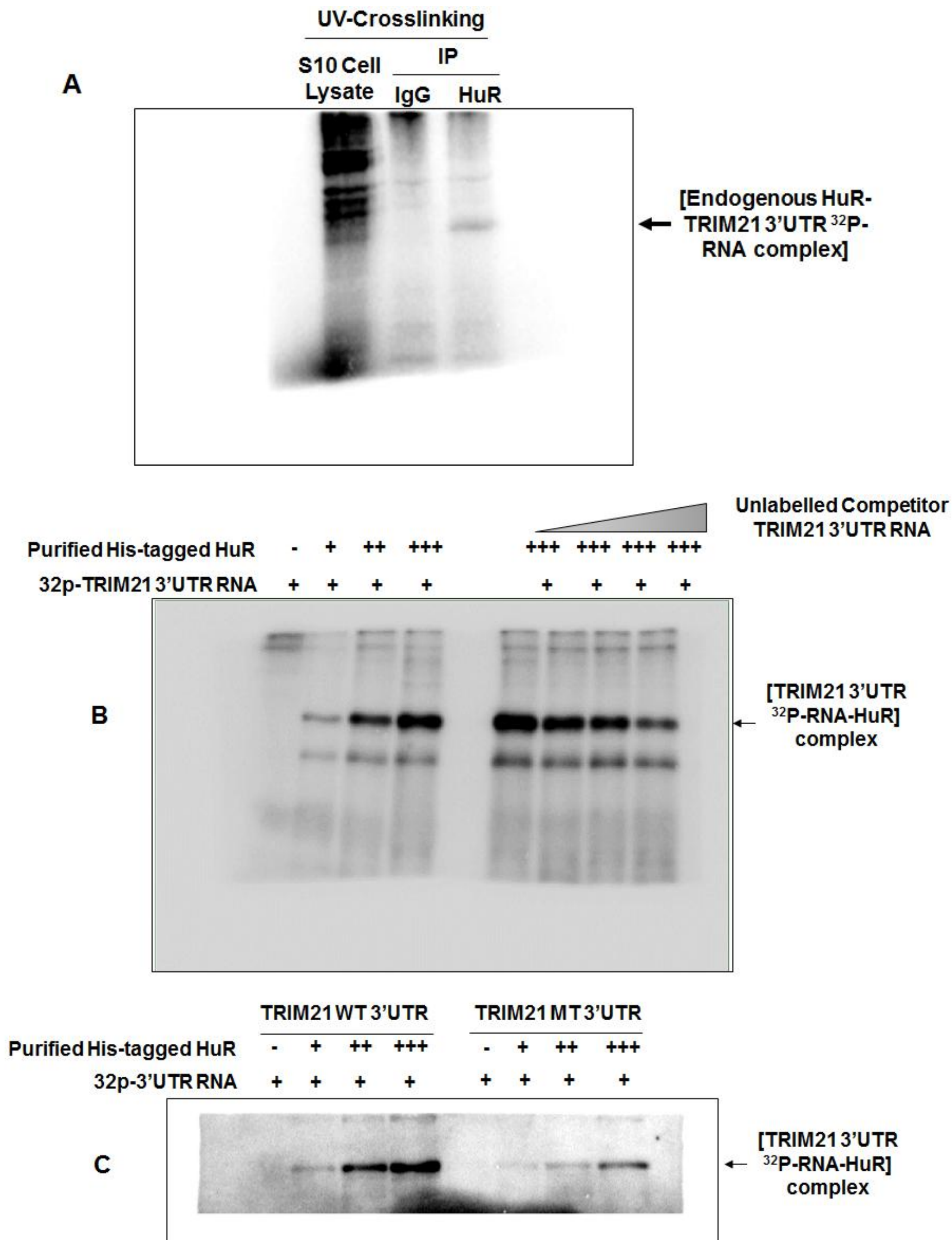

Supplementary Fig. 15. Full length scanned radioactive gel (SDS-PAGE) (uncropped) of (A) radioactive *TRIM21 3'UTR* (*WT*) and endo-HuR binding, (B) binding of radioactive *TRIM21 3'UTR* (*WT*) and purified 6x His-tagged HuR binding and (C) binding of radioactive *TRIM21 3'UTR (WT and MT*) with purified 6x His-tagged HuR binding shown in Fig. 4B, 4D, and 4E.

Fig. S16

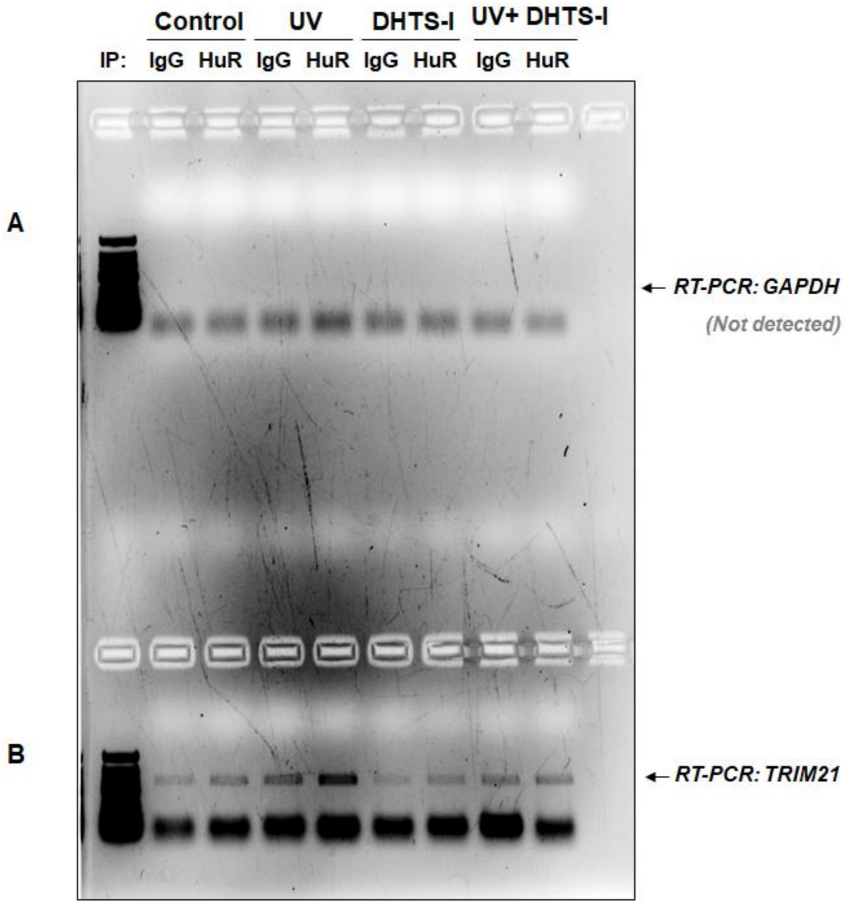

Supplementary Fig. 16. Full length ethidium bromide stained agarose gel (uncropped) of (B) *TRIM21* and (A) *GAPDH* shown in Fig. S7A.

Fig. S17

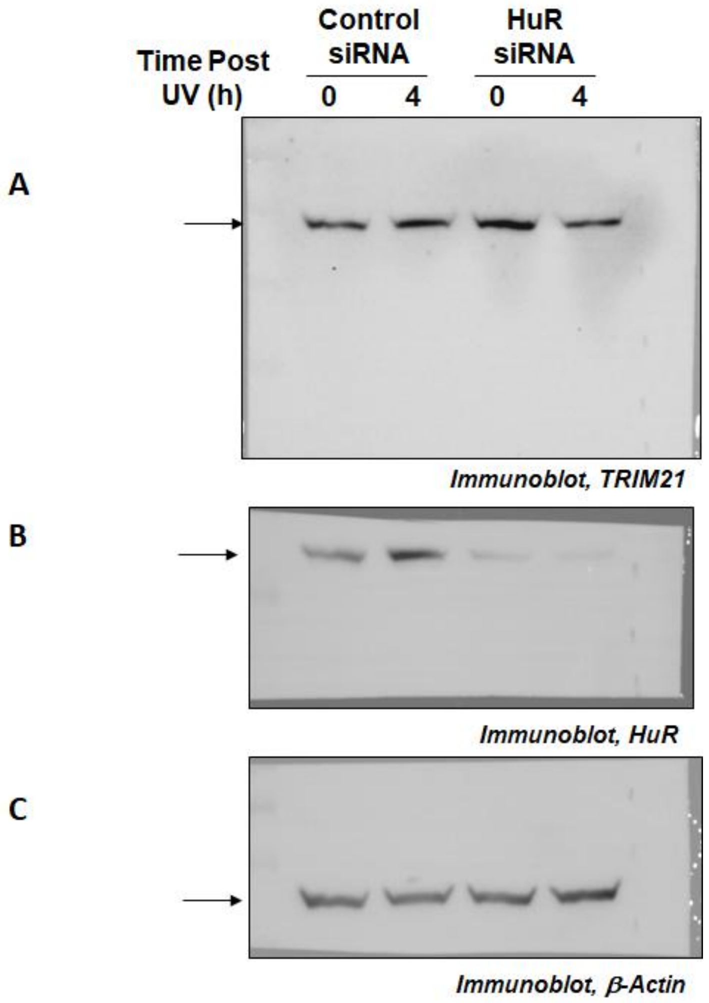

Supplementary Fig. 17. Full length blots (uncropped) of (A) TRIM21, (B) HuR, and (C) β-Actin shown in Fig. S7B.

Fig. S18

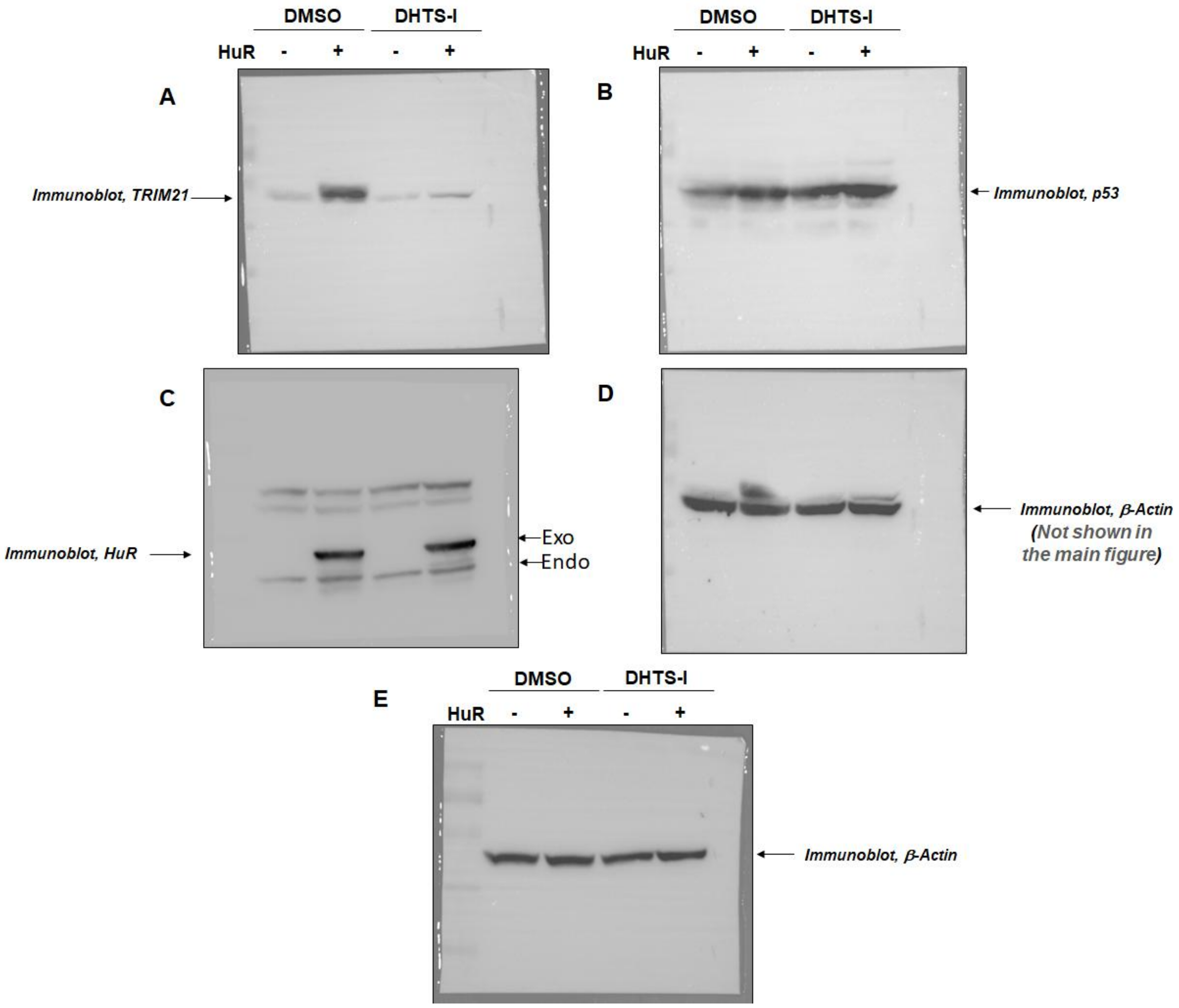

Supplementary Fig. 18. Full length blots (uncropped) of (A) TRIM21, (B) p53, (C) HuR, and (D and E) β-Actin shown in Fig. 6D. The upper band in (C) is of β-Actin in the unstripped blot reprobed with HuR antibody.

Fig. S19

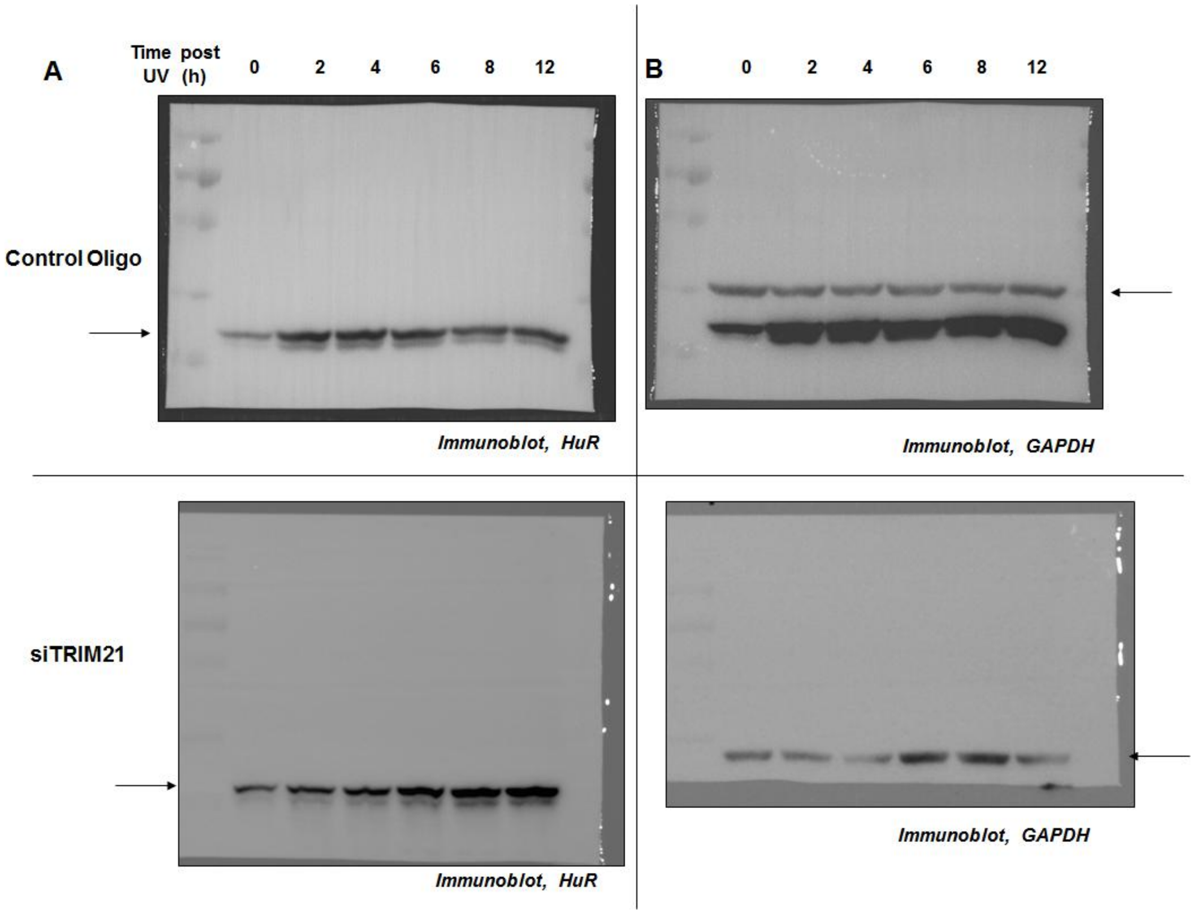

Supplementary Fig. 19. Full length blots (uncropped) of HuR (A), and GAPDH (B), shown in Fig. S1. The lower band in (B) is of HuR in the unstripped blot reprobed with GAPDH antibody.

Fig. S20

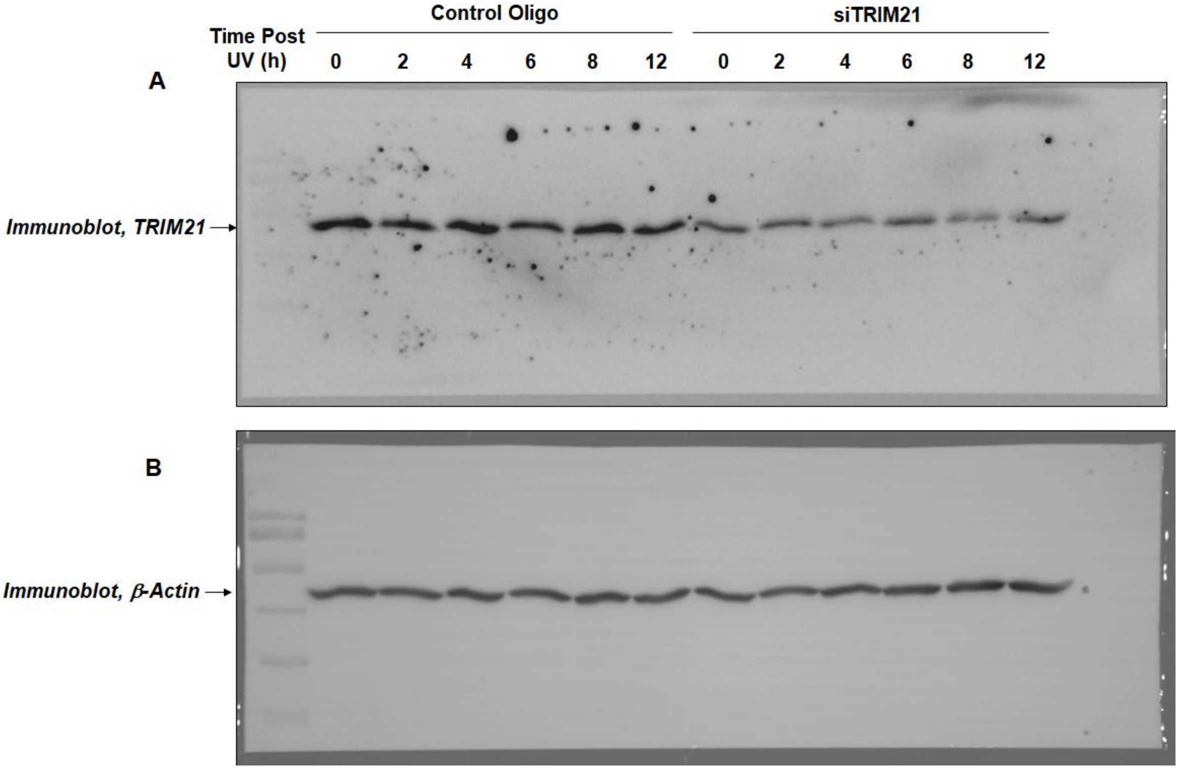

Supplementary Fig. 20. Full length blots (uncropped) of (A) TRIM21 and (B) β-Actin shown in Fig. S1.

Fig. S21

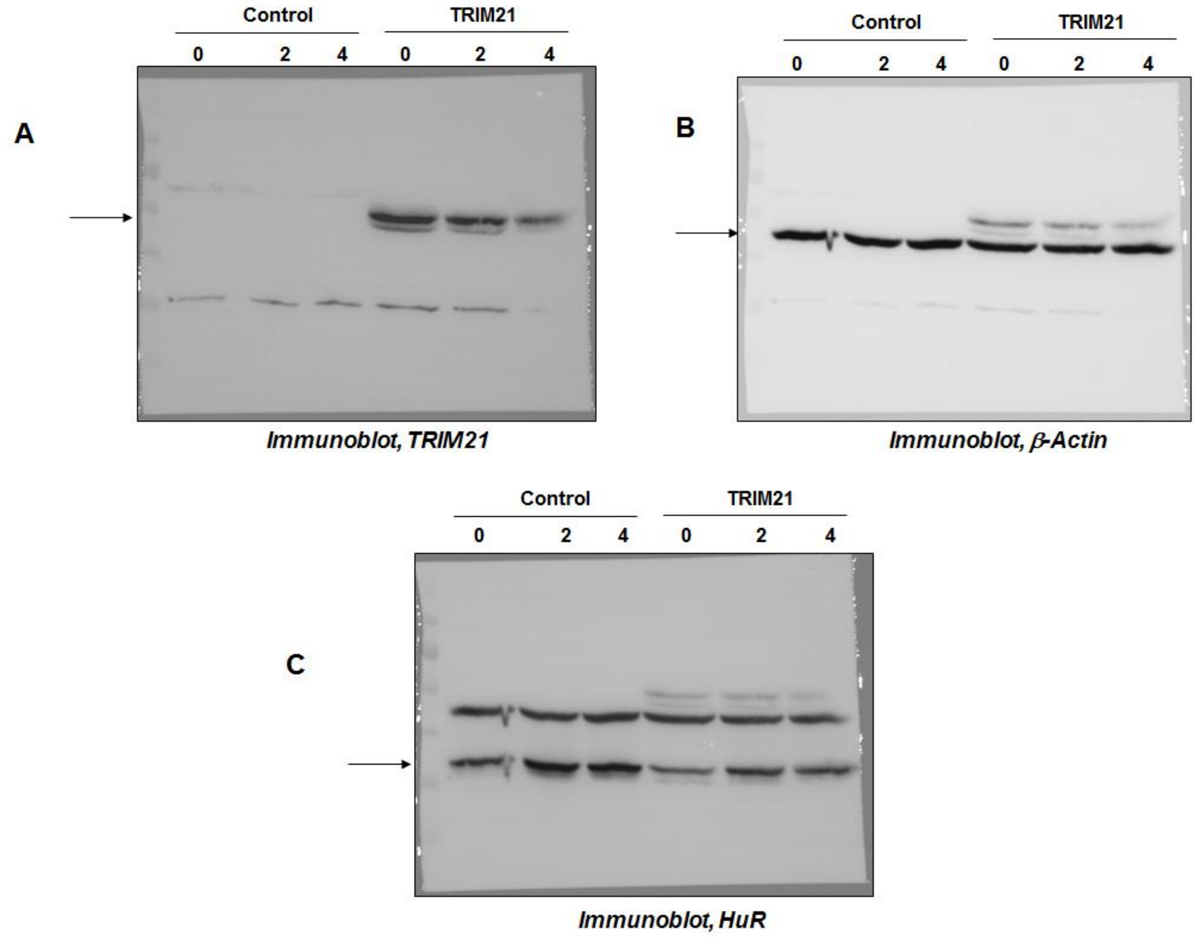

Supplementary Fig. 21. Full length blots (uncropped) of TRIM21 (A), β-Actin (B), and HuR (C) shown in Fig. S2. The upper band in (B) is of TRIM21 in the unstripped blot reprobed with Actin antibody and in (C) is of Actin in the unstrapped blot reprobed with HuR antibody.

Fig. S22

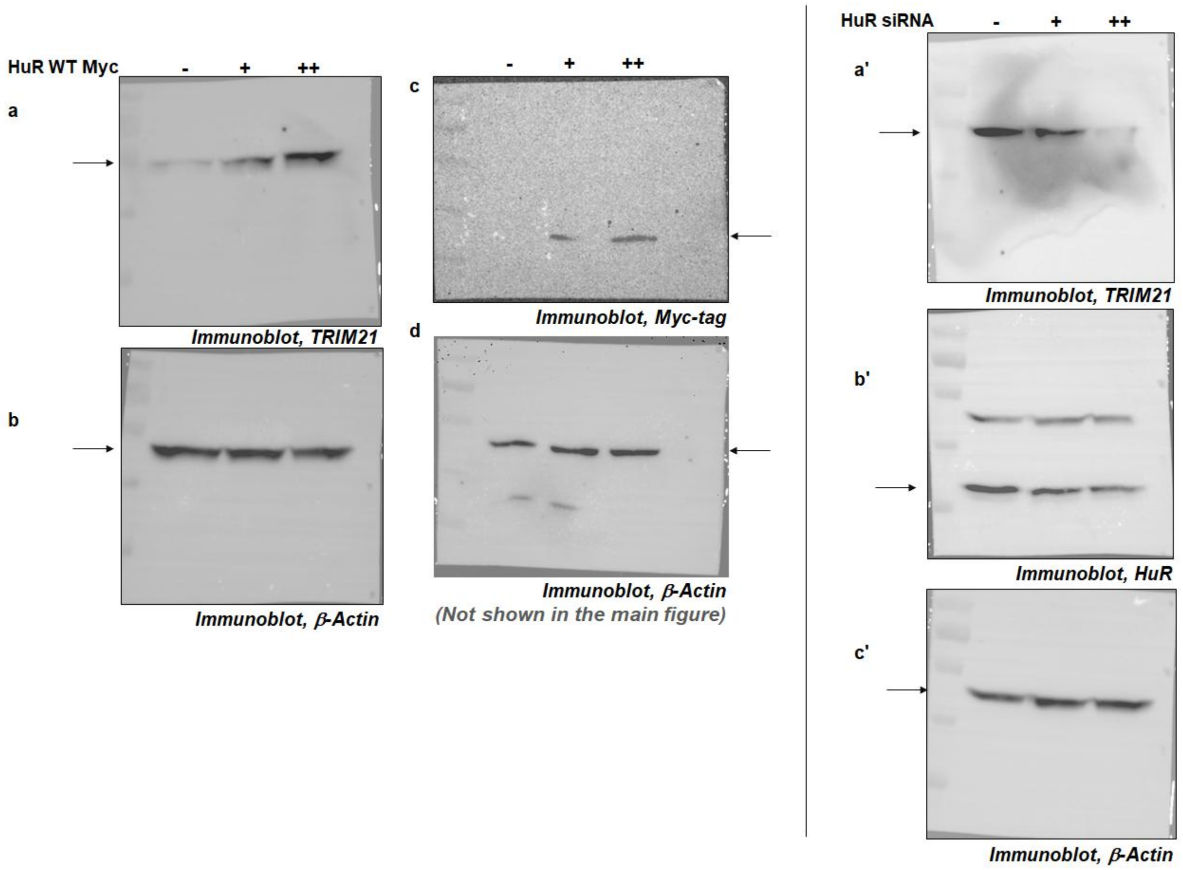

Supplementary Fig. 22. Full length blots (uncropped) of (a and a') TRIM21, (c) Myc-tag, (b') HuR and (b, d and c') β-Actin shown in Fig. S4A and S4B. The upper band in (b') is of β-Actin in the unstripped blot reprobed with HuR antibody.

Supplementary Methods

Primers sequences

No.	Name of Primer	Sequence of Primer (5’→3’)
1.	F Luc 800 F	CTCGGATATTTGATAGTTGG
2.	F Luc 900 R	GCGCACTTTGAATCATGTAA
3.	R Luc 1250 F	CATGTTGTGCCACATATTGA
4.	R Luc 1385 R	GTTCAAACCATGCAGTAAGAT
5.	GAPDH 840 F	TGATGACATCAAGAAGGTGGTGAAG
6.	GAPDH 1080 R	TCCTTGGAGGCCATGTGGGCCAT
7.	TRIM21 3’UTR EcoRI F	AGTGGAATTCTGGCTTTCTCTGGACACT
8.	TRIM21 3’UTR NotI R	ATATGCGGCCGCAGGAAGCCCTTTTGCA
9.	TRIM21 HuR BS mut F	ACTTAACGCGCACGCCACTCCTCTC
10.	TRIM21 Short F	ATTGTCCTGGAAAGGAGTGAGT
11.	TRIM21 Short R	CAGAGTGATGTGGACTGCACAT

siRNA targeting sequences

- siGENOME Non-Targeting siRNA Pool #1, D-001206-13-05 (Dharmacon)

  Target Sequence: Not provided by the manufacturer

- siGENOME SMARTpool, Human *ELAVL1* (1994), M-003773-04-0005 (Dharmacon)

  1. siGENOME SMARTpool siRNA D-003773-02, *ELAVL1*

     Target Sequence: GCAAUUACCAGUUUCAAUG

  2. siGENOME SMARTpool siRNA D-003773-04, *ELAVL1*

     Target Sequence: UCAAAGACGCCAACUUGUA

  3. siGENOME SMARTpool siRNA D-003773-05, *ELAVL1*

     Target Sequence: CAAAGACGCCAACUUGUAC

  4. siGENOME SMARTpool siRNA D-003773-21, *ELAVL1*

     Target Sequence: CGACUCAAUUGUCCCGAUA

- siGENOME SMARTpool, Human *TRIM21* (6737), M-006563-02-0005 (Dharmacon)

  1. siGENOME SMARTpool siRNA D-006563-06, *TRIM21*

     Target Sequence: UCUCAGAGCUAGAUCGAAG

  2. siGENOME SMARTpool siRNA D-006563-07, *TRIM21*

     Target Sequence: GAGCAUACCUGGAAAUGAA

  3. siGENOME SMARTpool siRNA D-006563-08, *TRIM21*

     Target Sequence: GGUGAUAAUUGUCCUGGAA

  4. siGENOME SMARTpool siRNA D-006563-09, *TRIM21*

     Target Sequence: AAGAGUGGCUUCUGGACAA
